# The identification of a N^6^-methyladenosin-modifed immune pattern to predict immunotherapy response and survival in urothelial carcinoma

**DOI:** 10.18632/aging.205782

**Published:** 2024-05-01

**Authors:** Xudong Mao, Xianjiong Chen, Zhehao Xu, Lifeng Ding, Wenqin Luo, Yudong Lin, Ruyue Wang, Liqun Xia, Mingchao Wang, Gonghui Li

**Affiliations:** 1 Department of Urology, Sir Run Run Shaw Hospital, Zhejiang University School of Medicine, Hangzhou 310016, China

**Keywords:** urothelial carcinoma, immune genes, prognosis, immunotherapeutic response, N^6^-methyladenosin, Cox proportional hazard model, random survival forest variable hunting, database analysis

## Abstract

Background: Dysregulation of the immune system and N^6^-methyladenosine (m6A) contribute to immune therapy resistance and cancer progression in urothelial carcinoma (UC). This study aims to identify immune-related molecules, that are m6A-modified, and that are associated with tumor progression, poor prognosis, and immunotherapy response.

Methods: We identified prognostic immune genes (PIGs) using Cox analysis and random survival forest variable hunting algorithm (RSF-VH) on immune genes retrieved from the Immunology Database and Analysis Portal database (ImmPort). The RM2Target database and MeRIP-seq analysis, combined with a hypergeometric test, assessed m6A methylation in these PIGs. We analyzed the correlation between the immune pattern and prognosis, as well as their association with clinical factors in multiple datasets. Moreover, we explored the interplay between immune patterns, tumor immune cell infiltration, and m6A regulators.

Results: 28 PIGs were identified, of which the 10 most significant were termed methylated prognostic immune genes (MPIGs). These MPIGs were used to create an immune pattern score. Kaplan-Meier and Cox analyses indicated this pattern as an independent risk factor for UC. We observed significant associations between the immune pattern, tumor progression, and immune cell infiltration. Differential expression analysis showed correlations with m6A regulators expression. This immune pattern proved effective in predicting immunotherapy response in UC in real-world settings.

Conclusion: The study identified a m6A-modified immune pattern in UC, offering prognostic and therapeutic response predictions. This emphasizes that immune genes may influence tumor immune status and progression through m6A modifications.

## INTRODUCTION

The advent of immune checkpoint blockade has markedly improved UC prognosis [1, 2]. Drugs like atezolizumab, durvalumab, avelumab, and nivolumab have received accelerated approval for UC’s second-line treatment [3]. Current immunotherapies primarily target T cells in the immune microenvironment. The immune checkpoint blockade lifts the suppression on T-cells, restoring their ability to attack and kill tumor cells, thereby curbing malignant tumor progression [4]. Despite this, studies indicate low positive response rates and emerging safety concerns, heightening the pharmaceutical industry’s interest in new immunotherapeutic targets [5, 6].

Increasing research reveals that m6A modification affects the response rate and drug resistance in immunotherapy [7, 8]. Aberrant m6A regulation often affects oncogenic and tumor-suppressing gene networks, modulating tumor immunogenicity, immune cell responses to tumors, and the tumor’s immune characteristics [9]. Research also shows that distinct m6A methylation patterns in the tumor microenvironment significantly influence the tumor immune regulatory network, impacting tumor initiation, metastasis, and drug resistance [10–13]. Here we have many reasons to assume that m6A methylation can modulate molecular components within the immune regulatory network, leading to transcriptome landscape reprogramming and alterations of cells in the tumor microenvironment, thereby influencing UC’s progression, prognosis, and resistance [10–13]. Identifying immune-related genes significantly modified by m6A is essential for the development of effective therapies combining anti-m6A strategies with immunotherapy.

Recent advancements in silicon-based methods, including machine learning and deep learning, have been instrumental in identifying biomarkers and molecular subtypes for diagnostic and prognostic purposes, and even in discovering new therapeutic targets [14]. This progress has helped to select patients who could maximize benefits while minimizing side effects and drug resistance [15]. Nevertheless, biomarker studies often suffer from inconsistency, mainly due to potential overfitting in silicon-based methods and the diverse nature of patient profiles [14], leading to issues in reproducibility and validation in independent datasets [16, 17].

Our study leveraged database analysis and machine learning technology to investigate molecular involvement in immune evasion. First, we retrieved immune-related molecules from the ImmPort [18] database, whose immune genes have been extensively researched in relation to the immune microenvironment, showing potential as immunotherapy targets. Then, by employing Cox proportional hazard models and an overfitting-resistant algorithm, RSF-VH [19], designed for high-dimensional variables like gene expression profiling, we identified an immune pattern significantly correlated with survival. To investigate whether prognostic immune-related genes indeed tend toward methylation, we utilized RM2Target [20], a comprehensive database for RNA modification targets, focusing on evidence from various experimental sources, to determine if immune genes of interest were significantly regulated by m6A modifiers. We also identified immune-related genes we were interested in with MeRIP-seq analysis, confirming their significant modification by m6A. Then, by ranking genes based on their importance values within this algorithm, we investigated the enrichment of m6A methylation in top importance immune genes. To ensure robust evidence, we explored this pattern’s associations with prognosis, progression, tumor immune cell microenvironment, and immune response in UC across multiple datasets.

## METHODS

### Materials

The microarray expression profile data from GSE32894 (*N* = 224), GSE32548 (*N* = 130), GSE13507 (*N* = 165), and GSE48075 (*N* = 73), along with demographic and clinical information, were downloaded from the Gene Expression Omnibus (http://www.ncbi.nlm.nih.gov/geo/) database. The TCGA-BLCA (*N* = 404) cohort data were obtained from The Cancer Genome Atlas (https://portal.gdc.cancer.gov). RNA-seq data of 298 advanced UC patients under atezolizumab monotherapy in the IMvigor210 trial, including best overall response (BOR) information, were sourced from published data [21]. Their corresponding clinical and survival information is summarized in Supplementary Table 1. Here, GSE32894 served as the training set for random survival forest analysis. GSE32894 was utilized as a training set for the machine learning method to uncover immune genes with the greatest impact on reducing survival in UC patients. TCGA-BLCA, GSE32548, GSE13507, and GSE48075 were employed as validation sets to confirm the clinical relevance of the identified key molecules within these datasets. Furthermore, the IMvigor210 dataset was used to explore whether the immune key molecules obtained could predict a patient’s response to immunotherapy in a real-world setting. The expression profiles in these datasets have all been standardized and normalized.

A comprehensive list of 1534 immune genes were downloaded from the ImmPort [18] database (https://immport.niaid.nih.gov). Experimental results from perturbation tests and MeRIP-seq were obtained from RM2Target (http://rm2target.canceromics.org/) for investigating immune gene regulation by writer, eraser and reader (WERs). 5 pairs of fastq files from bladder cancer samples undergoing MeRIP-seq were downloaded from the Sequence Read Archive (SRA) database under project PRJNA733602 for comparing the differences in m6A methylation levels among PIGs.

### RSF-VH and Cox proportional hazard model

Univariate Cox regression analysis identified prognostic immune-related genes (*p* < 0.01) as seed genes (Supplementary Table 2). These genes are further selected in the Cox weighted RSF-VH algorithm to select prognostic immune genes (PIGs). In RSF-VH, parameters were set as follows: “nsplit” to 10, “nrep” to 100, “nstep” to 5, growing 1000 trees with a k-value of 5, as previously recommended [22, 23]. 5-fold cross-validation was applied to prevent overfitting. Collinearity issues were addressed by estimating the condition number, ensuring no collinearities among selected variables (Supplementary Table 3).

### Validation of m6A methylation in immune genes

Using perturbation data from RM2Target, we intersected our immune genes of interest with those specifically detected for m6A methylation by the MeRIP-seq method. A hypergeometric distribution test was employed to determine whether genes deemed important in the random forest process were more likely to undergo m6A methylation by WERs. The top 10 most significant prognostic immune genes were termed methylated prognostic immune genes (MPIGs). The WER-MPIG relationships were integrated into a regulatory network using the “igraph” package and visualized accordingly. For the MeRIP-seq data from PRJNA733602, peak calling was performed with MACS2, followed by peak overlapping and merging using “DiffBind” package, and quantitative and differential analysis of these regions was conducted using “limma” and “edgeR” packages. Differential methylated sites between normal tissue samples and bladder cancer samples were identified using a threshold of *p* < 0.05.

### From nonlinear models to linear risk scoring

To address the poor variable interpretability of the nonlinear random forest model, a linear equation was employed:


∑i Coefficient (MPIGs)× Expression (MPIGs).

Using this formula, each patient’s risk score was calculated. The median risk score served as a cutoff to classify patients into high-risk and low-risk groups.

### Nomogram

A prognostic nomogram based on the MPIGs was established to predict the 1-year, 3-year, and 5-year disease-specific survival (DSS) in GSE32894, with calibration curves comparing model-predicted and actual DSS. The calibration curves for predicting the 3-year and 5-year DSS using the nomogram were generated with the “calibrate” package in R.

### Multi-dataset analysis

To obtain comprehensive and robust evidence, various statistical methods are employed to demonstrate the clinical relevance of MPIGs across multiple datasets, including GSE32894, GSE32548, GSE13507, GSE48075, and TCGA-BLCA. Kaplan-Meier analysis and log-rank test were employed to assess prognosis differences between risk groups. Patients’ death events were visualized on a risk score distribution using a dot plot. MPIGs were categorized as oncogenic or anti-cancer molecules, with their expression levels visualized using a heatmap across datasets. Univariate and multivariate Cox regression analyses determined the prognostic predictive ability of MPIGs and their independent predictive power for Overall Survival (OS) or DSS amidst other clinical factors. Seven Cox proportional hazards models using MPIGs predicted DSS in GSE13507, GSE32548, GSE32894, GSE48075, and OS in GSE13507, GSE48075, TCGA-BLCA datasets and their performances were evaluated using the concordance index (C-index). Violin plots with the Wilcoxon test showed associations between risk scores and clinical factors significantly affecting prognosis in univariate Cox regression analysis. Differential expression analysis between high-risk and low-risk groups explored the relevance between MPIGs and 24 well-known m6A methylation regulators.

### Comparison of MPIGs with classical immune checkpoints and potential therapeutic targets

Receiver operating characteristic curves (ROC curves) were used to determine the prognostic value of the risk score compared to several other classical immune checkpoints and therapeutic targets. In the ROC method, patients were divided into two subgroups based on survival: longer or shorter than the median DSS. Patients with survival shorter than the median DSS were excluded unless death had been observed.

### Immune infiltration analysis

The EPIC [24] program estimated proportions of various tumor immune microenvironment cells in GSE32894 dataset. The CIBERSORT algorithm [25] compared the proportions of immune-related cells of the same type as EPIC in high and low-risk groups of GSE32894 samples. The Estimate algorithm [26] was also applied to GSE32894 samples, calculating the Estimate score and Immune score for each patient with UC. The Immune score and the Estimate score are indicators used to assess the overall status of the tumor immune microenvironment. In simple terms, the Immune score is used to estimate the infiltration of immune cells in tumor tissues, while the Estimate score is used to assess the purity of the tumor. The abundance of cells in different risk groups was assessed using the Wilcoxon test to determine the differences.

### Real-world immune response analysis

In the IMvigor210 dataset of 298 advanced UC patients, patients were categorized into response (R for PR or CR) and non-response (NR for PD or SD) groups. Differential analyses were used to compare MPIG-derived risk scores between these groups. To validate that MPIG can predict the immune response rate of UC, the dataset was randomly split into a training set of 196 samples and a test set of 102 samples at a 2:1 ratio. Logistic regression was employed to model the response to atezolizumab in the training set, and the performance of the model was evaluated using ROC curves.

### Clinical samples and RNA extraction and real-time quantitative PCR

Bladder cancer specimens and matched adjacent normal tissues were obtained with patient consent from the Sir Run Run Shaw Hospital, School of Medicine, Zhejiang University. The study was approved by the Ethics Committee of the same institution (Ethical Approval Document Number 20200716-065). Patient demographics are shown in Supplementary Table 4. Total RNA from tissues was extracted using TRIzol reagent (Cwbiotech, China) following the manufacturer’s instructions. cDNA was synthesized using HiFiScript gDNA Removal RT MasterMix (Cwbiotech, China). RT-qPCR analysis was performed using 2× SYBR Green qPCR master mix (Cwbiotech, China) on a LightCycler^®^ 480 II System (Roche, Switzerland). Detailed primer sequences are listed in Supplementary Table 5.

### GSEA

R packages “edgeR” and “limma” identified differentially expressed genes between high-risk and low-risk groups in GSE32894. Then, GSEA analysis was used to explore KEGG pathway differences between risk groups. KEGG pathway signatures gene sets, “c2.cp.kegg.v7.4.symbols.gmt,” were used in GSEA, identifying enriched pathways with *p* < 0.01. Additionally, by utilizing immune cell gene sets that are broadly acknowledged in the field [27], we performed an ssGSEA analysis to ascertain the differences in enrichment levels of immune cell-related gene pathways among various risk groups delineated based on MPIGs. This analysis includes immune-related cell types consistent with the EPIC program, including CD4+ T cells, CD8+ T cells, macrophages, NK cells, and B cells.

### Summary of analytical tools

**Figure 1:**
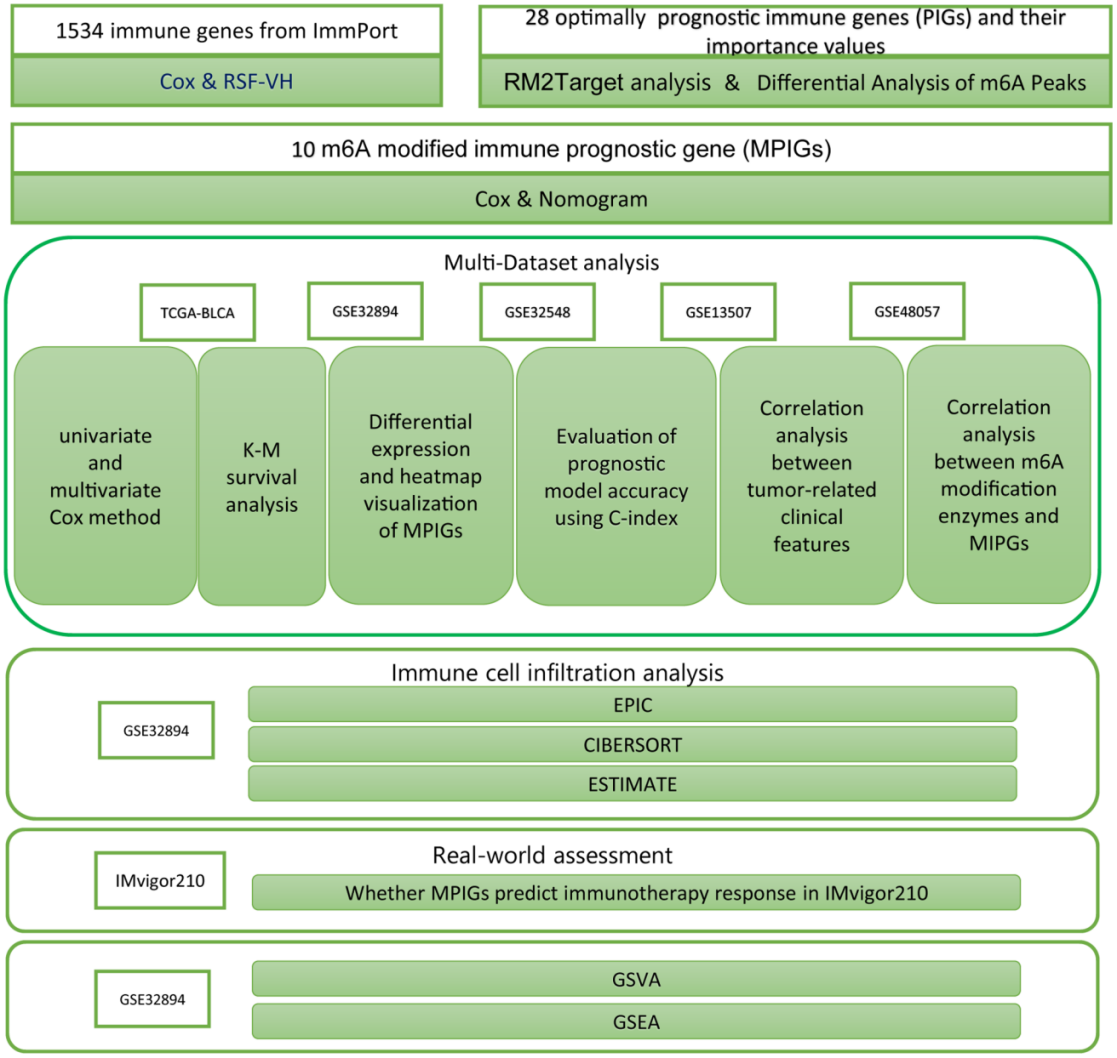
The workflow diagram delineates the methodologies and resources employed in this investigation.

Cox analysis and Kaplan-Meier analysis were conducted utilizing the “survival” package in R, and their results were visualized using the “survminer” package. Heatmaps in the article were generated using the “heatmap” package. The nomogram was created with the “regplot” package in R. Results from both univariate and multivariate Cox analyses were visualized using the “forestplot” package. The RSH-VH algorithm was implemented with the “randomForestSRC” package. ROC analysis was performed using the “pROC” package. The network diagram depicting relationships between m6A regulators and PIGs was constructed using the “igraph” package. EPIC analysis was conducted through the website https://epic.gfellerlab.org/. Estimate analysis was executed using the “CIBERSORT” package and, additionally, with the “estimate” package. GSEA was carried out using the “clusterProfiler” package and visualized using the “enrichplot” package. The ssGSEA analysis was conducted using the GSVA package, which calculates enrichment scores for different immune cell gene sets for each sample. Subsequently, a Wilcoxon test was employed to compare the means between different risk groups for hypothesis testing. The research procedures are summarized as a workflow, displayed in Figure 1.

### Availability of data and materials

The datasets utilized for this study can be accessed through public databases such as GEO (https://www.ncbi.nlm.nih.gov), TCGA (https://portal.gdc.cancer.gov/), SRA (https://www.ncbi.nlm.nih.gov/sra), and RM2Target (http://rm2target.canceromics.org/#/home). For any data, code and materials related to the research, please contact the corresponding author’s email, and we will make every effort to provide them.

## RESULTS

### Identification of the prognostic m6A-modified immune pattern

We initially identified a total of 166 candidate genes associated with prognosis in GSE32894 using the Cox proportional hazards model (*p* < 0.01; see Supplementary Table 2). Subsequently, the Cox weighted RSF-VH algorithm sequentially identified 28 immune genes significantly impacting the DSS of UC in GSE32894. These genes are INSR, NR1H3, CTSE, SDC1, PPARG, FAM3B, CXCL1, SPP1, PTHLH, IL24, TUBB3, CXCL2, TNFRSF6B, CYR61/CNN1, CTSB, STC2, VEGFC, PLCG1, SCG2, BMP1, CBL, MAP2K2, GRB2, CMTM1, SHFM1, PSMD2, CRLF1, and KLRC2, collectively termed as PIGs (see Supplementary Table 6).

**Figure 2:**
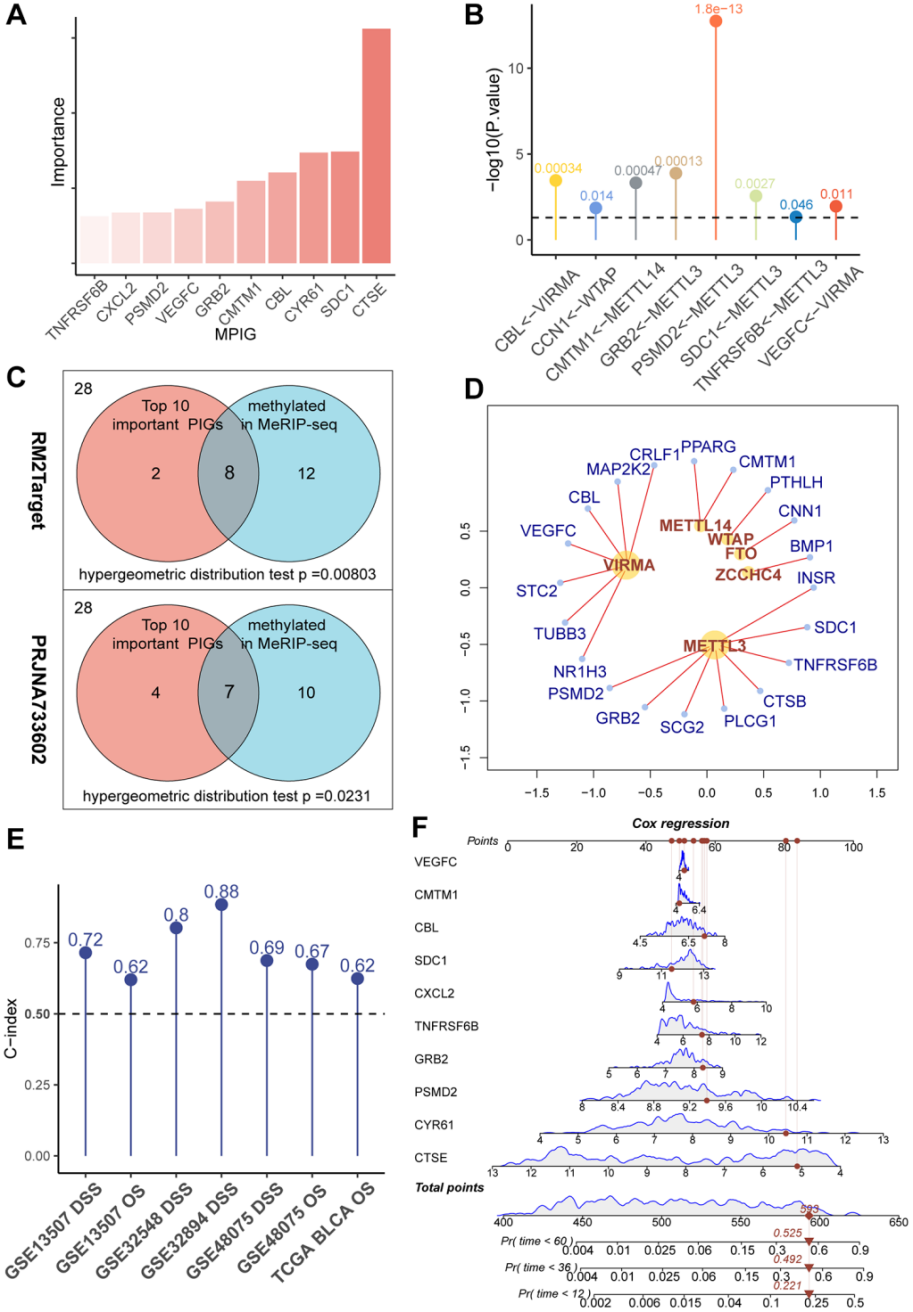
(**A**) A bar chart showcasing the “importance” of MPIGs arranged in descending order, derived from a random forest analysis. Greater importance of a variable indicates a larger discrepancy in prediction accuracy between models with and without the variable. (**B**) A balloon plot demonstrates significant regulatory relationships between WERs and corresponding MPIGs proven by perturbation experiments from RM2Target database. (**C**) Venn diagrams highlight that out of 28 PIGs identified in malignant cell lines from the RM2Target database through MeRIP-seq analysis, 20 exhibit significant m6A methylation modifications. Among the top 10 PIGs (MPIGs) for importance, 8 are methylated. Hypergeometric distribution tests suggest a propensity for m6A methylation in PIGs associated with prognosis. Further, in the PRJNA733602 dataset, 7 out of the top 10 most important PIGs show enhanced m6A peak differences, underscoring a correlation between prognostic relevance and increased m6A modification levels. (**D**) A network diagram reveals the regulatory relationships between WERs and their regulated PIGs within the RM2Target database. (**E**) Balloon plots display the predictive performance of Cox models constructed with MPGs for UC prognosis across multiple datasets, as indicated by the C-index. (**F**) A nomogram drawn from Cox models based on MPGs in the GSE32894 dataset, illustrating individual patient scores and their corresponding survival probabilities.

To clarify the correlation between gene expression levels and the prognosis of UC, we introduced the concept of ‘importance’ in random forests. In essence, the greater the importance of a gene, the more accurate the forest model, including that gene, is in predicting the prognosis compared to a forest model without it. After ranking PIGs in descending order based on ‘importance’ values, CTSE, PSMD2, CXCL2, CYR61, CMTM1, VEGFC, SDC1, TNFRSF6B, GRB2, and CBL were considered particularly crucial in the composition of the random forest model (Figure 2A). In subsequent studies, we used these 10 genes to construct an immune prognostic pattern.

### m6A modification tendency increases with the prognostic relevance of PIGs in malignant cell lines and cancer tissues of UC

We retrieved 28 PIGs in the MeRIP-seq data from RM2Target, of which 20 were detected to be methylated at the m6A site. Among the top 10 genes considered as the most important, 8 exhibited m6A methylation modifications (Figure 2B). Based on these findings, we conducted a hypergeometric distribution test and discovered that m6A modification was more likely to occur in the “more important” top 10 genes (*p* = 0.00584 Figure 2C). This indicates that m6A modified immune genes are more deeply involved in the prognosis of UC patients than those immune genes that are unmodified or modified at low levels.

Through the utilization of the RM2Target database, for the 28 PIGs we identified, we discovered 20 pairs of significant regulatory associations between PIGs and m6A regulators. These associations were then organized into a network, presented in Figure 2D, and detailed in Supplementary Table 7. In this network, we observed that METTL3 and VIRMA covered the most PIGs and exerted significant regulatory effects on them. Notably, ongoing clinical trials are currently focusing primarily on METTL3 and VIRMA [28]. Our data reveal the pivotal roles of METTL3 and VIRMA within the immune-related m6A modification process, suggesting that the design of inhibitors targeting METTL3 and VIRMA may represent a promising avenue for integrating anti-m6A therapy with immunotherapy.

In PRJNA733602, we first applied MACS2 to call peaks on the MeRIP-seq files of 5 paired bladder cancer and normal tissues. Subsequently, we utilized DiffBind to overlap and merge the peaks. Quantitative and differential analyses of these peak regions were performed using limma and edgeR. A total of 32,801 gene modification sites were analyzed, with a significance threshold set at *p* < 0.05. Among them, 2,747 gene modification sites were identified as exhibiting significant differences in m6A modification peaks. Among all 28 PIGs, 17 genes were found to have elevated m6A modification levels in tumors (see Supplementary Table 8). In the top 10 PIGs, 7 genes, namely CXCL2, PSMD2, GRB2, CMTM1, CBL, SDC1, and CTSE, showed increased m6A modification levels. Through a hypergeometric distribution test, we observed that genes with greater importance in the random survival forest tended to exhibit more pronounced differences in methylation levels between bladder cancer samples and normal tissues (*p* = 0.0231, Figure 2C). Consequently, we referred to the top 10 important PIGs as methylated prognostic immune Genes (MPIGs).

### MPIGs formulate precise prediction of UC prognosis

Subsequently, we employed MPIGs to construct Cox proportional hazards models separately in the GSE3289, GSE32548, GSE13507, GSE48075, and TCGA-BLCA datasets. The prognostic capacity of MPIGs was assessed using the C-index. Remarkably, across all datasets, irrespective of using OS or DSS as the prediction outcome, the C-index of the Cox model built with MPIGs consistently surpassed 0.5, indicating a high and stable prediction accuracy (see Figure 2E). Particularly in independent cohorts GSE32548 and GSE13507 the model achieved accuracies exceeding 0.8 and 0.7, respectively. Furthermore, we established a nomogram for predicting DSS in the GSE32894 utilizing MPIGs, and the calibration curve affirmed its prognostic predictive performance (see Figure 2F and Supplementary Figures 1 and 2).

**Figure 3:**
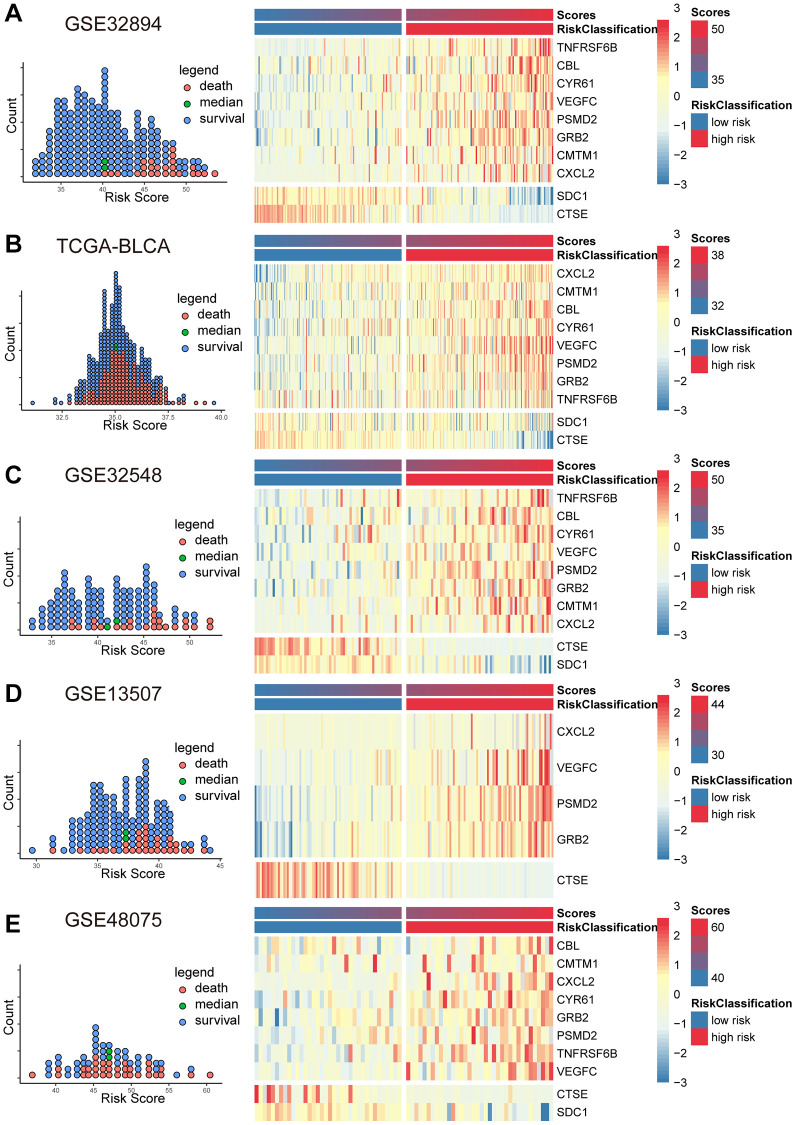
The scatter plots (**A**–**E**) on the left illustrate the distribution of death events across risk scores derived from MPIGs. The heatmap (**A**–**E**) on the right displays the specific upregulation or downregulation of MPIGs expression across different risk groups.

Based on the coefficients derived from the Cox model and the expression levels of MPIGs, we calculated the risk scores for each patient in GSE32894, TCGA-BLCA, GSE32548, GSE13507, and GSE48075. Scatter plot shows that patients incline to have higher mortality with a risk score above the median for the cohort, and the heat map illustrates that patients with higher risk score obviously tend to express a higher level of risky MPIGs (TNFRSF6B, CXCL2, PSMD2, VEGFC, GRB2, CMTM1, CBL, and CYR61) in their samples of tissue, whereas patients with lower risk score were more likely to express a higher level of suppressor MPIGs (here were SDC1 and CTSE) (Figure 3A–3E). Moreover, the direction of upregulation or downregulation of MPIGs was consistent across different data sets.

**Figure 4:**
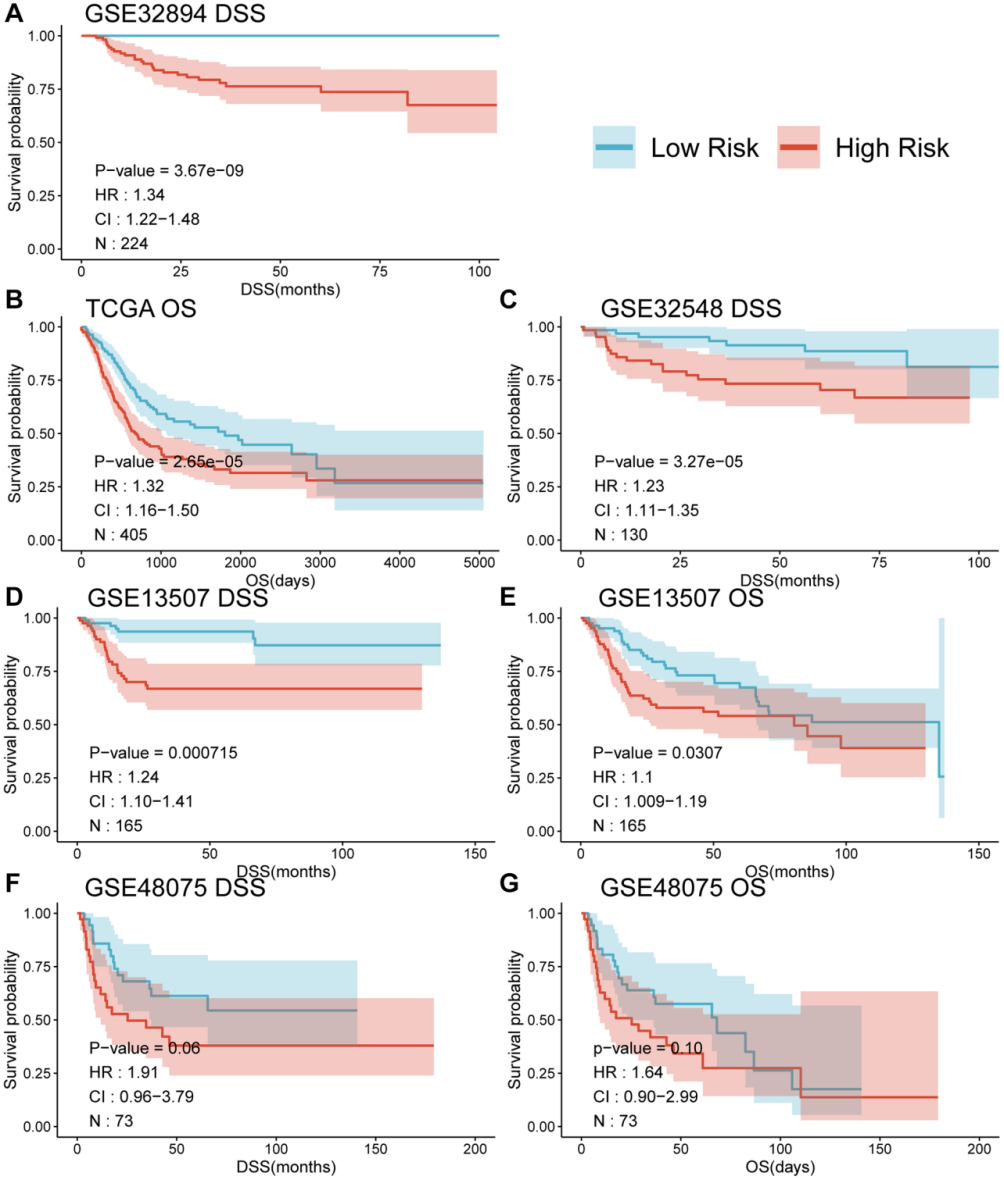
Survival curves (**A**–**G**) comparing the survival between different risk groups based on MPIGs across various datasets.

When comparing the DSS differences between high and low-risk groups using Kaplan-Meier survival analysis across multiple datasets, a notable separation of survival curves was evident, with significant *p*-values observed in GSE32894 (*p* < 0.0001), GSE32548 (*p* = 0.01), and GSE13507 (*p* = 0.00021) for DSS, and in TCGA-BLCA (*p* = 0.00036) and GSE13507 (*p* = 0.045) for OS among UC patients in distinct risk groups based on their risk score. Although GSE48075 showed a *p*-value of 0.06 for DSS and 0.1 for OS, distinct survival curves were still observable (refer to Figure 4, panels A, C, D, F for DSS; B, E for OS; and G for GSE48075 OS analysis). Simultaneously, employing the univariate Cox proportional hazards model, the risk score derived from MPIGs was characterized, revealing a significant correlation between this risk score and diminished survival in UC across all datasets, except for GSE48075. Specifically, in GSE32894, the HR for DSS was estimated at 1.34 (95% CI: 1.22–1.48, *p* < 0.001); in TCGA-BLCA, the HR for OS was also estimated at 1.32 (95% CI: 1.16–1.50, *p* < 0.001); in GSE32548, the HR for DSS was 1.23 (95% CI: 1.11–1.35, *p* < 0.001); in GSE13507, the HR for DSS was 1.24 (95% CI: 1.10–1.41, *p* < 0.001); and in GSE13507, the HR for OS was 1.1 (95% CI: 1.09–1.19, *p* = 0.031).

**Figure 5:**
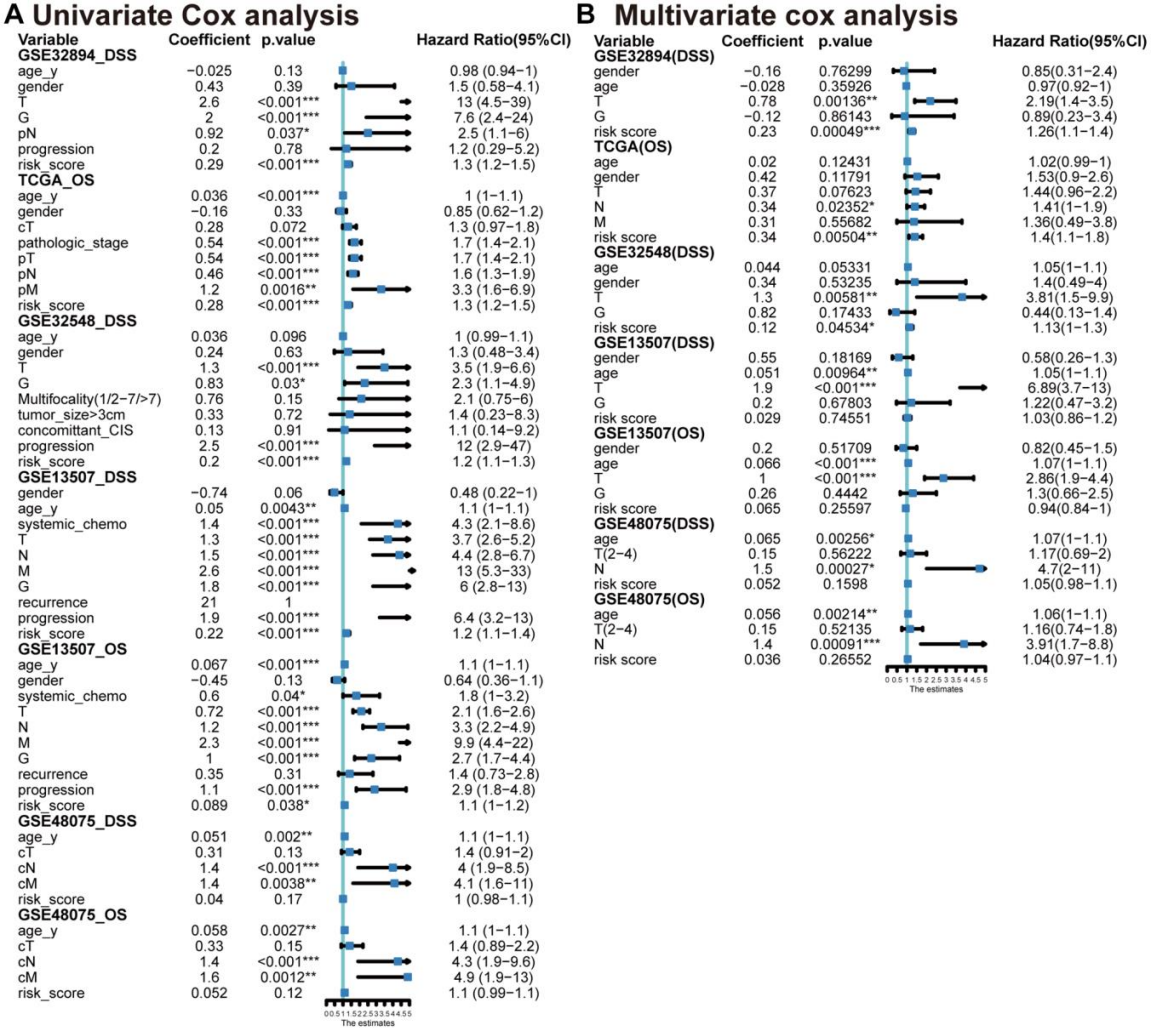
Univariate (**A**) and multivariable (**B**) Cox analyses conducted across various datasets.

Incorporating significant prognostic risk factors identified through the univariate Cox model (Figure 5A), we integrated them with the risk score in the multivariate Cox model. This analysis revealed that the MPIGs-derived risk score serves as an independent risk factor for reduced survival in UC, a finding consistently validated across multiple datasets (Figure 5B). Specifically, in GSE32894, the hazard ratio (HR) for the risk score was 1.26 (95% CI: 1.10–1.40, *p* = 0.00049). Similarly, in TCGA-BLCA, the HR was 1.4 (95% CI: 1.10–1.80, *p* = 0.00504). In GSE32548, the HR was 1.13 (95% CI: 1.00–1.30, *p* = 0.0453).

### MPIGs correlate to prognostic clinical factors in UC

**Figure 6:**
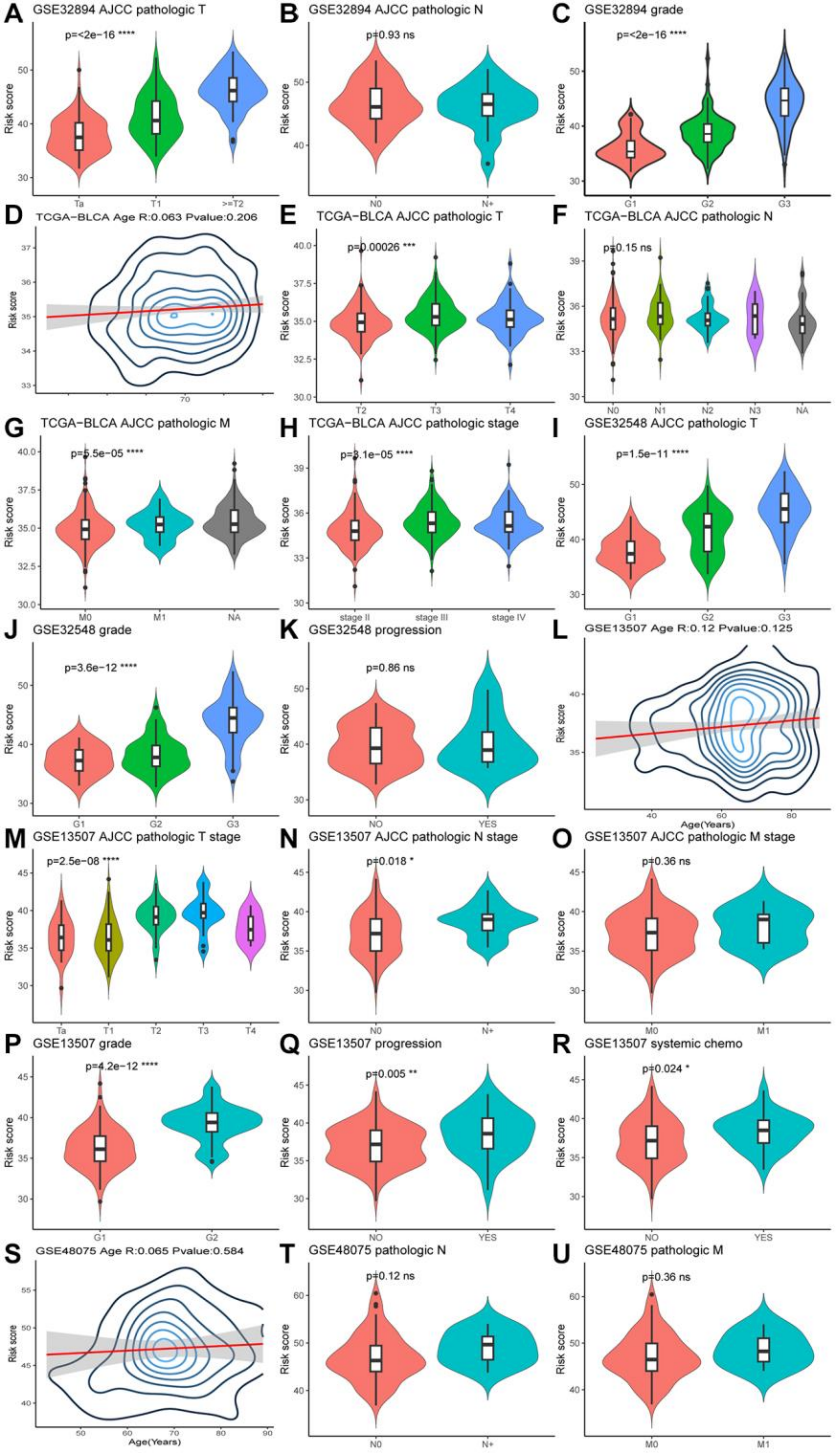
Violin plots (**A**–**U**) illustrate the relationship between clinical factors and risk scores, employing the Wilcoxon test for comparisons between two variables and the Kruskal-Wallis test for comparisons among more than two variables.

The Wilcoxon test was used to examine the association between MPIGs and significant prognostic risk factors identified in the previous univariate Cox analysis. In GSE32894, patients with advanced T stage and G grade exhibited higher risk scores, as depicted in Figure 6A, 6C. Similarly, in the TCGA-BLCA cohort, higher risk scores correlated with advanced pathologic T stage, M stage, and pathological stage, illustrated in Figure 6E, 6G, 6H. Figure 6I, 6J in GSE32548 demonstrated a positive relationship between risk scores and advanced T stage and G grade. In GSE13507, Figure 6M, 6N, 6P, 6Q, 6R showed that patients at higher risk tended to have advanced T stage, N stage, G grade, a higher likelihood of progression, and were more likely to receive chemotherapy. Conversely, violin plots in Figure 6B, 6F did not reveal an association between risk score and pathological N stage in the GSE32894 and TCGA-BLCA cohorts. Similarly, M stage and clinic N stage were not significantly associated with risk scores in GSE13507 and GSE48075, as shown in Figure 6O, 6T, 6U. Additionally, patient age showed no correlation with MPIGs across the cohorts (Figure 6D, 6L, 6S). Notably, in GSE32548, no correlation was observed between the risk score and tumor progression in non-muscle invasive bladder cancer samples (Figure 6K).

### MPIGs correlate to immune cell infiltration in the tumor microenvironment in UC

**Figure 7:**
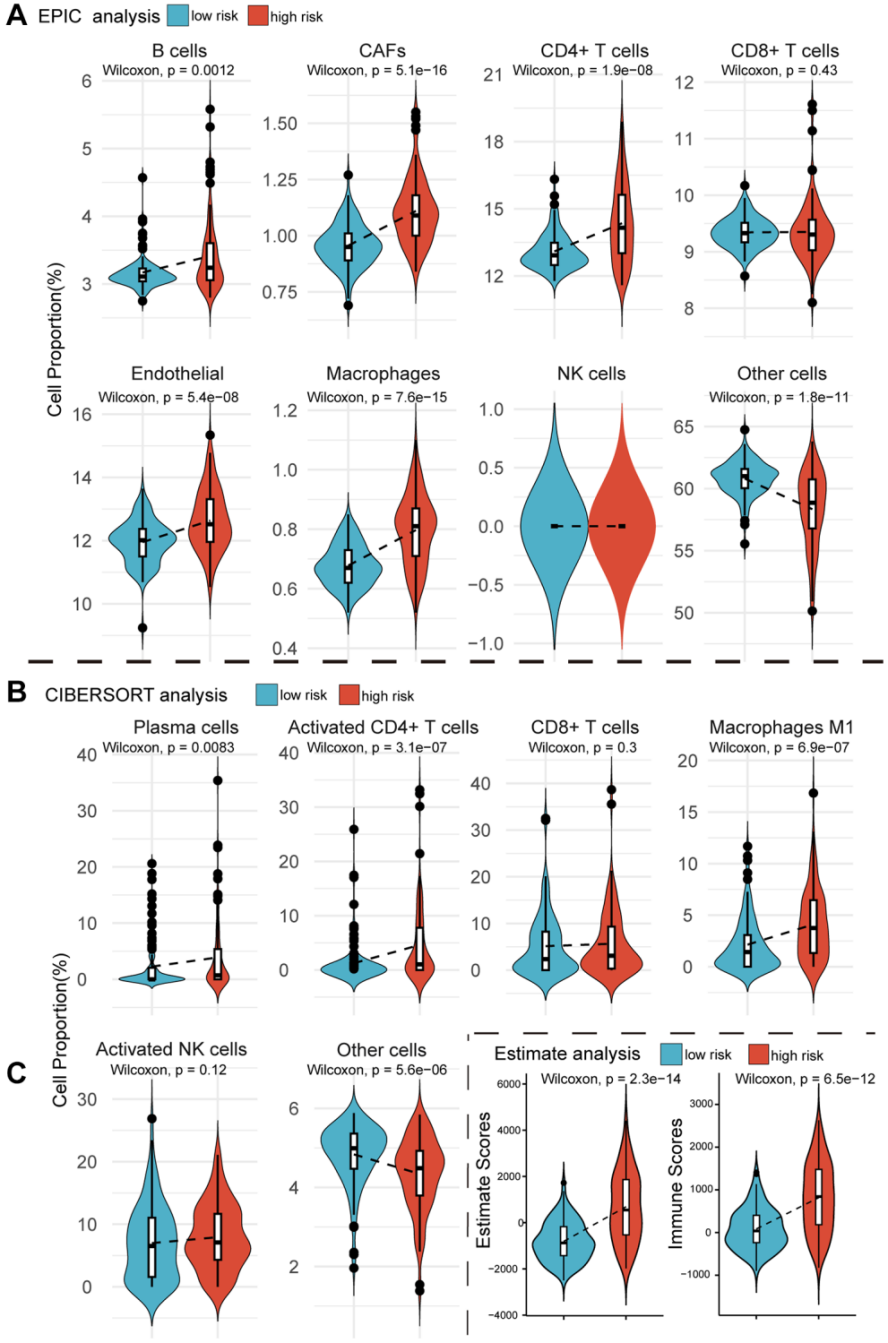
**Violin plots reveal the results of cellular infiltration analyses within the GSE32894 expression matrix.** (**A**) The EPIC web tool estimates infiltration scores for B cells, CAFs, CD4+ T cells, CD8+ T cells, endothelial cells, macrophages, NK cells, and other cells in UC tumors from GSE329894, comparing between high and low risk groups based on MPIGs. (**B**) The CIBERSORT algorithm calculates infiltration scores for immune cells, including plasma cells, activated CD4 T cells, CD8 T cells, macrophages M1, activated NK cells, and other cells involved in EPIC, with comparisons between high and low risk groups derived from MPIGs. (**C**) Estimate analysis calculates Estimate scores and Immune scores for each patient in GSE32894, comparing between high- and low-risk groups derived from MPIGs.

If MPIGs can accurately identify the immune status characterized by significant infiltration of immune cells, it can be considered indicative of their potential to predict whether a patient is suitable for receiving immune therapy [29]. We here employed three computational algorithms, namely EPIC, CIBERSORT, and Estimate, to evaluate the immune cell infiltration status for different risk groups of tumors in GSE32894. EPIC analysis explored the correlation between MPIGs and the tumor microenvironment. As depicted in Figure 7A, the proportions of infiltrating B cells (*p* = 0.0012), cancer-associated fibroblasts (CAFs) (*p* = 5.1e−16), CD4+ T cells (*p* = 1.9e−08), endothelial cells (*p* = 5.4e−08), and macrophages (*p* = 7.6e−15) significantly increased in the high-risk group compared to the low-risk group. However, no significant differences were observed in the abundance of infiltrating CD8+ T cells and NK cells between the low- and high-risk groups. To validate the EPIC-derived conclusions regarding immune cell involvement in tumor microenvironments, we employed CIBERSORT to assess the infiltration abundances of plasma cells, activated CD4+ T cells, CD8+ T cells, M1 macrophages, and activated NK cells within GSE32894 tumor samples. Comparisons of the infiltration were subsequently made between different risk groups.

Figure 7B illustrates that the proportions of infiltrating plasma cells (*p* = 0.0083), activated CD4+ T cells (*p* = 3.1e−07), and macrophages (*p* = 6.9e−07) were significantly higher in the high-risk group compared to the low-risk group. Additionally, CIBERSORT analysis did not reveal any differences in the proportions of infiltrating CD8+ T cells and NK cells between the two groups. We observed a significant agreement between the outcomes derived from EPIC analysis and CIBERSORT analysis, both suggesting that MPIGs can accurately discern the immune infiltration status of tumors. This variation was predominantly driven by the differential infiltration of CD4+ T cells, B cells, and macrophages, while CD8+ T cells, although showing no difference in infiltration between high and low-risk subgroups, still exhibited substantial overall infiltration. Finally, we introduced the Estimate algorithm to calculate Immune scores and Estimate scores for each UC patient in GSE32894, comprehensively characterizing their immune infiltration depth and tumor purity. Upon comparing these scores between the high and low-risk groups, we found that the high-risk group exhibited significantly higher immune infiltration depth (*p* = 6.5e−12) and tumor purity (*p* = 2.3e−14) than the low-risk group (Figure 7C).

### Investigation of the relationship between m6A regulators and MPIGs across multiple datasets

**Figure 8:**
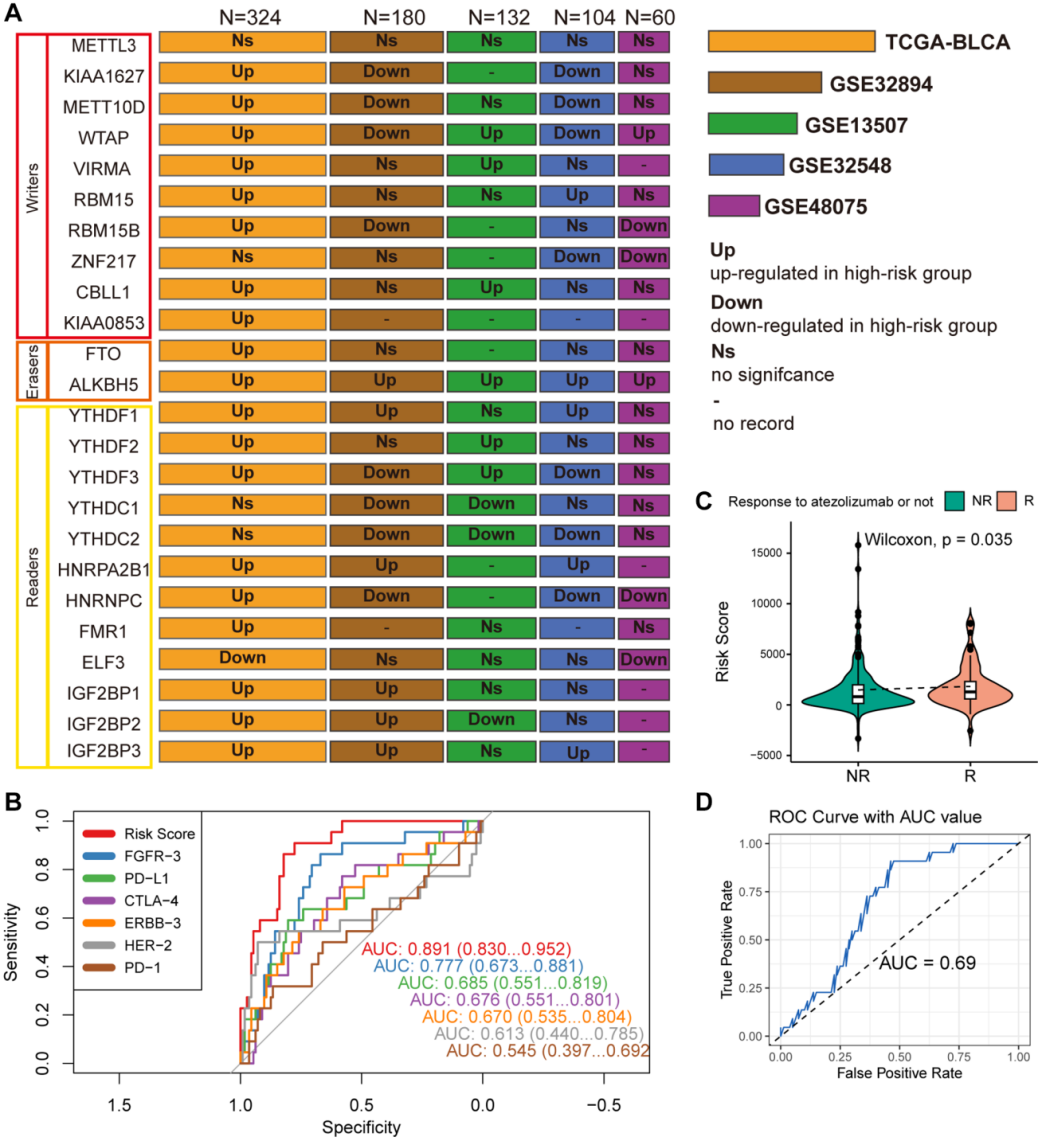
(**A**) Wilcoxon test compares differences in the expression of 24 widely reported WERs between different risk groups across datasets GSE32894, GSE13507, GSE48075, and TCGA-BLCA, summarizing significantly upregulated or downregulated findings with box plots. (**B**) ROC curves compare the performance of MPIG-derived risk scores and the expression levels of FGFR-3, PD-L1, CTLA-4, ERBB-3, HER-2, PD-1 in predicting survival shortening events in UC patients. (**C**) Violin plots and the Wilcoxon test compare differences in MPIG-derived risk scores between responders and non-responders to monotherapy immunotherapy (atezolizumab) in the IMvigor210 trial. (**D**) ROC curves and AUC values assess the performance of a binary model predicting the response of advanced UC patients to immunotherapy, based on MPIG-derived risk scores, tested in a random external dataset.

Differential expression analysis was conducted on 24 widely reported m6A regulators across multiple datasets, comparing their expression patterns between distinct risk groups defined by MPIGs. These 24 m6A regulators encompass methyltransferases (writers: METTL3, KIAA1627, METT10D, WTAP, VIRMA, RBM15, RBM15B, ZNF217, CBLL1, KIAA0853), demethylases (erasers: FTO, ALKBH5), and binding proteins (readers: YTHDF1, YTHDF2, YTHDF3, YTHDC1, YTHDC2, HNRPA2B1, HNRNPC, FMR1, ELF3, IGF2BP1, IGF2BP2, IGF2BP3). The results from each cohort were collected and summarized in Figure 8A.

In the TCGA-BLCA cohort, with the largest sample size, most m6A regulators (19/24) exhibited upregulated expression in the high-risk group, except for ELF3, which demonstrated lower expression. Particularly noteworthy was the significantly upregulated expression of the eraser ALKBH5 in the high-risk groups across all included datasets. However, an unexpected finding emerged, revealing no significant expression difference of METTL3 between the two risk groups in all five independent datasets. While KIAA1627 and METT10D displayed high expression levels in TCGA-BLCA, their expression levels were lower in the high-risk group of other two datasets (GSE32894 and GSE32548). Elevated expression levels of VIRMA and CBLL1 were observed in the high-risk group TCGA-BLCA and GSE13507. RBM15 showed significant upregulation in the high-risk group in TCGA-BLCA and GSE32548. YTHDF1, HNRPA2B1, and IGF2BP3 exhibited high expression in high-risk patients across TCGA-BLCA, GSE32548, and GSE13507. Conversely, although there was no significant difference in expression in TCGA-BLCA, YTHDC1 demonstrated marked downregulation in GSE32894 and GSE13507. Similarly, YTHDC2 displayed lower expression levels in the high-risk group in GSE32894, GSE13507, and GSE32548. In summary, m6A regulators demonstrated aberrant expression in at least one independent cohort.

### Compare MPIGs with potential therapeutic targets

As illustrated in Figure 8B, the ROC curves, which compare MPIGs with six extensively studied therapeutic targets and immune checkpoints (FGFR, PDL1, CTLA4, ERBB3, HER2, PD1), reveal that MPIGs achieved the highest area under the curve (AUC) value, reaching an estimated 0.871. This result substantiates the stability and reliability of MPIGs in predicting survival outcomes.

### MPIGs predicts response to immunotherapy in UC in real world setting

298 patients with advanced UC who received atezolizumab as first-line treatment were divided into a response group, referring to complete response and partial response, and a non-response group, referring to stable disease and progressive disease. The expression levels of MPIGs were used to calculate risk scores for each patient, and the Wilcoxon test was performed to compare the differences between the groups. Patients who responded to atezolizumab had lower risk scores compared to non-responding patients (*p* = 0.035, Figure 8C). This suggests that patients identified as high-risk by MPIGs are more likely to develop resistance to immune therapy. The 298 samples were randomly divided into two groups in a 2:1 ratio, and logistic regression models were trained using the risk score as a variable and response/non-response as the outcome in the larger dataset. The model was tested in the training set and evaluated using ROC analysis, with an area under the curve of 0.69. The optimal cutoff value had a specificity of 0.64 and sensitivity of 0.73 (Figure 8D).

### Aberrant expression of MPIGs in tumor tissue and its association with disease-free survival in UC

**Figure 9:**
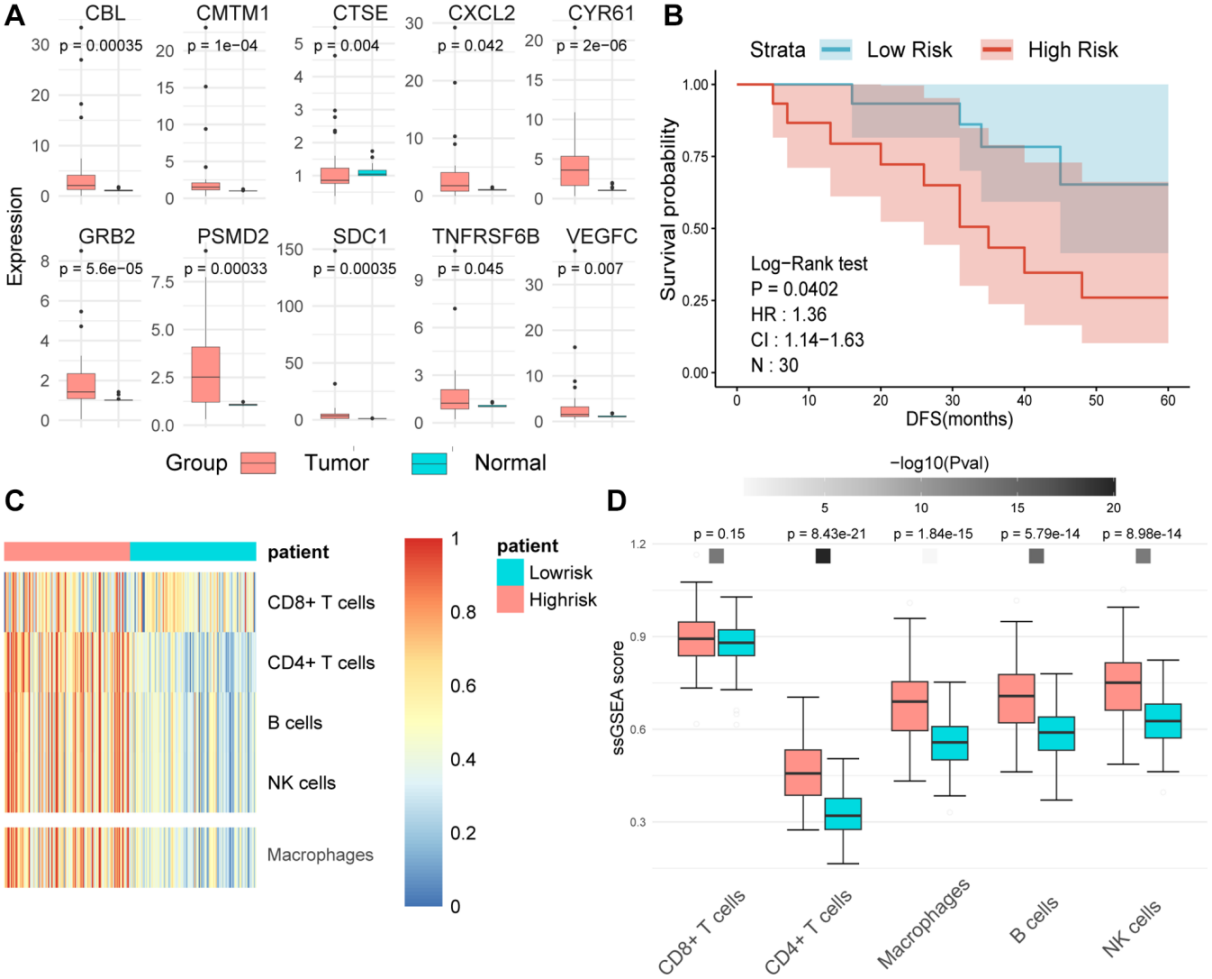
(**A**) The expression levels of tumors and adjacent normal tissues were evaluated using real-time quantitative PCR. Split-panel box plots contrast the differences in expression levels of various MPIGs between the two types of tissues. (**B**) Kaplan-Meier analysis and survival curves reveal the disparity in disease-free survival (DFS) among 30 urothelial carcinoma (UC) patients recruited by Shao Yifu Hospital, categorized into high- and low-risk groups based on MPIGs. (**C**) A heatmap illustrates the infiltration scores of CD8+ T cells, CD4+ T cells, B cells, NK cells, and macrophages in GSE32894 samples, calculated using the ssGSEA method. (**D**) Box plots compare the differences in infiltration scores of CD8+ T cells, CD4+ T cells, B cells, NK cells, and macrophages between high- and low-risk groups derived from MPIGs.

We employed RT-qPCR technology to evaluate the expression levels of MPIGs in UC tissue samples and matched adjacent normal tissues obtained from our hospitals. The RT-qPCR analysis unveiled heightened expression levels of all 10 MPIGs within the tumor mass of UC, demonstrating a significant increase compared to the adjacent normal tissue (Figure 9A). Disease-free survival (DFS) data were collected for all clinical samples, and based on the RT-qPCR results quantifying MPIGs expression, risk scores were computed for each UC case, leading to the stratification into high and low-risk groups. Subsequently, Kaplan-Meier analysis delineated distinctly separated survival curves for patients with different risk profiles (*p* = 0.0407, Figure 9B). Additionally, univariate Cox regression analysis suggested a significant inverse correlation between risk scores as a continuous variable and reduced DFS in UC (HR: 1.36, 95% CI: 1.14–1.64, *p* = 0.000651).

### GSEA analysis

To gain deeper insights into the biological pathways associated with our MPIGs-derived risk scores, the expression data obtained from GSE32894 was stratified based on ranking scores. Subsequent differential expression analysis was conducted to identify genes exhibiting significant expression differences between the delineated groups. Following this, GSEA analysis was performed to identify biological pathways that demonstrated significant enrichment. The five most significantly enriched pathways include: including PATHWAYS_IN_CANCER, CELL_ADHESION_MOLECULES_CAMS, MELANOGENESIS, LYSOSOME, and INSULIN_SIGNALING_PATHWAY (see Supplementary Figure 3). Remarkably, the enriched pathways associated with MPIGs predominantly involve cancer-related processes. This observation suggests a substantial correlation between the malignance of UC patients and the MPIGs prognostic panel we developed.

Further, employing ssGSEA on the GSE32894 gene expression matrix, with the gene sets identified by Gabriela Bindea et al. for recognizing various immune cells, revealed that pathways related to CD4+ T cells, macrophages, B cells, and NK cells were significantly upregulated in the high-risk group derived from MPIGs [29]. Conversely, CD8+ T cell pathway levels did not show notable differences between the risk groups derived from MPIGs (refer to Figure 9C, 9D). Intriguingly, this conclusion aligned with our findings from the EPIC analysis and CIBERSORT, strongly suggesting that the MPIGs-derived risk scores can distinguish between two tumor immune microenvironments characterized by different cell types, including CD4+ T cells, B cells, and macrophages.

## DISCUSSION

m6A stands as the predominant modification type in RNA-level epigenetic alterations, significantly correlated with the progression, metastasis, and prognosis of bladder urothelial carcinoma. Importantly, ongoing researches have unveiled the association between m6A modification and the immune status of cancer, potentially impacting the efficacy of immunotherapy [9, 30–32]. Given the low response rates to immunotherapy in UC and the limited robustness of molecular predictors like PD1/PDL1 in gauging immunotherapeutic responses [33, 34], we utilized bioinformatics approaches to validate a novel set of m6A-modified immune genes. Through this endeavor, we unveiled their biological significance in the context of UC progression and treatment, thus highlighting their potential as targets for anti-m6A therapy and combination immunotherapy.

Prominent biomarkers of biological significance require the capability to exhibit consistency across various cohorts [35]. To attain prognostic molecules with generalizability, we initially employed anti-overfitting machine learning tools, specifically RSF-SH combined with Cox regression analysis, within public database datasets. Through this approach, we identified a set of 28 immune genes significantly associated with UC prognosis. Given the relatively large panel of these 28 genes, we prioritized the top 10 genes based on their “importance” parameter to construct a more “compact” model, thereby enhancing model generalizability. Subsequently, the clinical relevance of these 10 genes was validated across 5 independent datasets to ensure absence of overfitting.

Previous studies have predominantly focused on RNA molecules and proteins significantly associated with m6A regulators [36, 37], even the m6A regulators themselves [38]. Given that m6A represents a novel regulatory factor in the immune system [39], our research aims to elucidate immune genes significantly modified by m6A. We posit that this approach is essential for establishing a bridge between immunity, m6A regulation, and cancer prognosis. It is well-known that by modifying specific RNA in various types of cancer, endogenous m6A regulates the fate of tumor cells in this manner [32, 40]. In our study, we demonstrated that the stronger the correlation between immune genes and shortened prognosis in (UC), the higher the likelihood of these genes undergoing m6A methylation we termed these prognosis-associated immune genes prone to m6A methylation as MPIGs. The risk scores formed by MPIGs correlate with increased infiltration of tumor-associated fibroblasts, macrophages, CD4+ T cells, and B cells (plasma cells) within UC. Furthermore, risk scores derived from MPIGs were not only significantly associated with survival time in UC patients in the real world but also found to predict the response rate of UC patients to immunotherapy. Our data provide relatively substantial evidence for such a hypothesis: in UC, m6A directly regulates the RNA metabolism of immune genes through methylation, accelerating disease progression, altering tumor immune cell infiltration status, and influencing tumor response to immunotherapy during progression. Our findings underscore the relationship between m6A and the tumor immune environment, particularly highlighting the interaction of our identified MPIGs—specifically TNFRSF6B, CXCL2, PSMD2, VEGFC, GRB2, CMTM1, CBL, CYR61, SDC1, and CTSE—with upstream m6A regulators, primarily METTL3 and VIRMA, significantly affecting UC progression. Our study not only suggests that the risk scores they form can serve as biomarkers for predicting immune response rates, but also supports their potential as targets for anti-m6A therapy and combination immunotherapy.

In our investigation, we observed that the “hot” tumor immune infiltration status predicted by MPIGs manifests as a tumor microenvironment primarily characterized by differential immune cell populations, including CD4+ T cells, B cells, and macrophages. Additionally, we noted that the proportion of CD8+ T cell infiltration within the tumor immune microenvironment did not differ significantly among risk groups derived from MPIGs, but the absolute proportion of T cell infiltration remained notably high. Nowadays, the body’s anti-tumor immune response is primarily mediated by tumor-specific CD8+ T cells [41]. The majority of clinically applied tumor immunotherapies rely on the ability of CD8+ T cells to directly recognize and eliminate tumor cells [42]. However, therapeutic strategies revolving around CD8+ T cells are constrained by the emergence of tumor cells with MHC defects and the formation of an immunosuppressive tumor microenvironment [43]. In various solid tumors, including bladder cancer, approximately 20–60% of tumor immune evasion is attributed to MHC-I defects and decreased recognition capacity of CD8+ T cells [44, 45]. Increasing evidences underscore the substantial role of synergy between CD4+ T cells and B cells in combating tumors. Studies also report cooperative actions between helper T cells and macrophages, mobilizing the entire immune arsenal against cancer cells [46]. Craft and Joshi’s team found that B cells presenting tumor antigens can promote the differentiation of tumor-specific helper CD4+ T cells, producing IL-21, enhancing anti-tumor CD8+ T cell responses, and thereby controlling tumor growth [47]. Kruse and colleagues discovered in animal models that helper T cells sometimes could more effectively resist cancer cells than killer T cells and eliminated cancer cells missed by killer T cells [46]. Moreover, m6A regulatory enzymes, especially writers and erasers, influence the proliferation and infiltration of CD4+ T cells in the tumor immune microenvironment [48]. Zheng et al. reported the reduction of mRNA m6A methylation in developing B cells severely blocks B cell development in mice [49]. Our study indicates that m6A modification significantly affects the immune status of bladder urothelial carcinoma, where the collaboration between CD4+ T cells and B cells may play a crucial role. Targeting therapies for this disease should primarily focus on CD4+ T and B cells to achieve more targeted effects.

This study’s limitations include a relatively small overall sample size despite confirming the clinical relevance of MPIGs across multiple datasets. Laboratory experiments validated by RT-qPCR used limited sample sizes, and the available survival data from donors only encompass DFS, lacking comprehensive metrics such as OS and DSS. Further research is needed to delineate the specific impact of m6A modifications on MPIGs on the progression, immune status, and resistance mechanisms in bladder urothelial carcinoma.

## CONCLUSION

We used machine learning techniques to avoid overfitting in public databases, and identified an immune pattern associated with reduced survival in UC. We found that the more immune genes associated with reduced survival in bladder urothelial carcinoma, the more likely they are to be m6A modified. We identified 10 so-called MPIGs, and believed they could serve as key bridging genes linking tumor immunity, m6A modification, and UC progression, potentially becoming new targets for anti-m6A therapy and combination immunotherapy.

## Supplementary Materials

Supplementary Figures

Supplementary Table 1

Supplementary Table 2

Supplementary Tables 3-6 and 8

Supplementary Table 7

## References

[1] Feld E, Harton J, Meropol NJ, Adamson BJS, Cohen A, Parikh RB, Galsky MD, Narayan V, Christodouleas J, Vaughn DJ, Hubbard RA, Mamtani R. Effectiveness of First-line Immune Checkpoint Blockade Versus Carboplatin-based Chemotherapy for Metastatic Urothelial Cancer. Eur Urol. 2019; 76:524–32. 10.1016/j.eururo.2019.07.032 31362898 PMC6822167

[2] Balar AV. Immune Checkpoint Blockade in Metastatic Urothelial Cancer. J Clin Oncol. 2017; 35:2109–12. 10.1200/JCO.2017.72.8444 28481707

[3] Parikh RB, Galsky MD, Gyawali B, Riaz F, Kaufmann TL, Cohen AB, Adamson BJS, Gross CP, Meropol NJ, Mamtani R. Trends in Checkpoint Inhibitor Therapy for Advanced Urothelial Cell Carcinoma at the End of Life: Insights from Real-World Practice. Oncologist. 2019; 24:e397–9. 10.1634/theoncologist.2019-0039 30944183 PMC6656487

[4] Kamada T, Togashi Y, Tay C, Ha D, Sasaki A, Nakamura Y, Sato E, Fukuoka S, Tada Y, Tanaka A, Morikawa H, Kawazoe A, Kinoshita T, et al. PD-1^+^ regulatory T cells amplified by PD-1 blockade promote hyperprogression of cancer. Proc Natl Acad Sci U S A. 2019; 116:9999–10008. 10.1073/pnas.1822001116 31028147 PMC6525547

[5] Chen X, Xu R, He D, Zhang Y, Chen H, Zhu Y, Cheng Y, Liu R, Zhu R, Gong L, Xiao M, Wang Z, Deng L, Cao K. CD8^+^ T effector and immune checkpoint signatures predict prognosis and responsiveness to immunotherapy in bladder cancer. Oncogene. 2021; 40:6223–34. 10.1038/s41388-021-02019-6 34552192

[6] Patel VG, Oh WK, Galsky MD. Treatment of muscle-invasive and advanced bladder cancer in 2020. CA Cancer J Clin. 2020; 70:404–23. 10.3322/caac.21631 32767764

[7] Shriwas O, Mohapatra P, Mohanty S, Dash R. The Impact of m6A RNA Modification in Therapy Resistance of Cancer: Implication in Chemotherapy, Radiotherapy, and Immunotherapy. Front Oncol. 2021; 10:612337. 10.3389/fonc.2020.612337 33718113 PMC7947626

[8] Zhang Z, Zhang C, Luo Y, Wu P, Zhang G, Zeng Q, Wang L, Yang Z, Xue L, Zheng B, Zeng H, Tan F, Xue Q, et al. m^6^A regulator expression profile predicts the prognosis, benefit of adjuvant chemotherapy, and response to anti-PD-1 immunotherapy in patients with small-cell lung cancer. BMC Med. 2021; 19:284. 10.1186/s12916-021-02148-5 34802443 PMC8607595

[9] Li X, Ma S, Deng Y, Yi P, Yu J. Targeting the RNA m^6^A modification for cancer immunotherapy. Mol Cancer. 2022; 21:76. 10.1186/s12943-022-01558-0 35296338 PMC8924732

[10] Cao X, Geng Q, Fan D, Wang Q, Wang X, Zhang M, Zhao L, Jiao Y, Deng T, Liu H, Zhou J, Jia L, Xiao C. m^6^A methylation: a process reshaping the tumour immune microenvironment and regulating immune evasion. Mol Cancer. 2023; 22:42. 10.1186/s12943-022-01704-8 36859310 PMC9976403

[11] Zhang L, Dou X, Zheng Z, Ye C, Lu TX, Liang HL, Wang L, Weichselbaum RR, He C. YTHDF2/m^6^ A/NF-κB axis controls anti-tumor immunity by regulating intratumoral Tregs. EMBO J. 2023; 42:e113126. 10.15252/embj.2022113126 37345898 PMC10390869

[12] Li D, Liu Y, Zhou J, Chen Y, Yang C, Liu H, Li W, You J. m6A Regulator-mediated RNA Methylation Modulates Immune Microenvironment of Hepatitis B Virus-related Acute Liver Failure. Inflammation. 2023; 46:1777–95. 10.1007/s10753-023-01841-2 37256461

[13] Ge J, Liu SL, Zheng JX, Shi Y, Shao Y, Duan YJ, Huang R, Yang LJ, Yang T. RNA demethylase ALKBH5 suppresses tumorigenesis via inhibiting proliferation and invasion and promoting CD8^+^ T cell infiltration in colorectal cancer. Transl Oncol. 2023; 34:101683. 10.1016/j.tranon.2023.101683 37224767 PMC10302567

[14] Vidotto T, Nersesian S, Graham C, Siemens DR, Koti M. DNA damage repair gene mutations and their association with tumor immune regulatory gene expression in muscle invasive bladder cancer subtypes. J Immunother Cancer. 2019; 7:148. 10.1186/s40425-019-0619-8 31174611 PMC6556053

[15] Sarfaty M, Golkaram M, Funt SA, Al-Ahmadie H, Kaplan S, Song F, Regazzi A, Makarov V, Kuo F, Ostrovnaya I, Seshan V, Zhao C, Greenbaum B, et al. Novel Genetic Subtypes of Urothelial Carcinoma With Differential Outcomes on Immune Checkpoint Blockade. J Clin Oncol. 2023; 41:3225–35. 10.1200/JCO.22.02144 36927002 PMC10256354

[16] Su Q, Wu H, Zhang Z, Lu C, Zhang L, Zuo L. Exosome-Derived Long Non-Coding RNAs as Non-Invasive Biomarkers of Bladder Cancer. Front Oncol. 2021; 11:719863. 10.3389/fonc.2021.719863 34490118 PMC8417445

[17] Shariat SF, Lotan Y, Vickers A, Karakiewicz PI, Schmitz-Dräger BJ, Goebell PJ, Malats N. Statistical consideration for clinical biomarker research in bladder cancer. Urol Oncol. 2010; 28:389–400. 10.1016/j.urolonc.2010.02.011 20610277 PMC3407571

[18] Bhattacharya S, Andorf S, Gomes L, Dunn P, Schaefer H, Pontius J, Berger P, Desborough V, Smith T, Campbell J, Thomson E, Monteiro R, Guimaraes P, et al. ImmPort: disseminating data to the public for the future of immunology. Immunol Res. 2014; 58:234–9. 10.1007/s12026-014-8516-1 24791905

[19] Ishwaran H, Kogalur UB, Chen X, Minn AJ. Random survival forests for high-dimensional data. Stat Anal Data Min. 2011; 4:115–32. 10.1002/sam.10103

[20] Bao X, Zhang Y, Li H, Teng Y, Ma L, Chen Z, Luo X, Zheng J, Zhao A, Ren J, Zuo Z. RM2Target: a comprehensive database for targets of writers, erasers and readers of RNA modifications. Nucleic Acids Res. 2023; 51:D269–79. 10.1093/nar/gkac945 36300630 PMC9825529

[21] Balar AV, Galsky MD, Rosenberg JE, Powles T, Petrylak DP, Bellmunt J, Loriot Y, Necchi A, Hoffman-Censits J, Perez-Gracia JL, Dawson NA, van der Heijden MS, Dreicer R, et al, and IMvigor210 Study Group. Atezolizumab as first-line treatment in cisplatin-ineligible patients with locally advanced and metastatic urothelial carcinoma: a single-arm, multicentre, phase 2 trial. Lancet. 2017; 389:67–76. 10.1016/S0140-6736(16)32455-2 27939400 PMC5568632

[22] Ishwaran H, Kogalur UB, Gorodeski EZ, Minn AJ, Lauer MS. High-dimensional variable selection for survival data. J Am Stat Assoc. 2010; 105:205–17. 10.1198/jasa.2009.tm08622

[23] Ishwaran H, Kogalur UB. Random survival forests for R. R news 7. 2007; 25–31.

[24] Racle J, Gfeller D. EPIC: A Tool to Estimate the Proportions of Different Cell Types from Bulk Gene Expression Data. Methods Mol Biol. 2020; 2120:233–48. 10.1007/978-1-0716-0327-7_17 32124324

[25] Chen B, Khodadoust MS, Liu CL, Newman AM, Alizadeh AA. Profiling Tumor Infiltrating Immune Cells with CIBERSORT. Methods Mol Biol. 2018; 1711:243–59. 10.1007/978-1-4939-7493-1_12 29344893 PMC5895181

[26] Yoshihara K, Shahmoradgoli M, Martínez E, Vegesna R, Kim H, Torres-Garcia W, Treviño V, Shen H, Laird PW, Levine DA, Carter SL, Getz G, Stemke-Hale K, et al. Inferring tumour purity and stromal and immune cell admixture from expression data. Nat Commun. 2013; 4:2612. 10.1038/ncomms3612 24113773 PMC3826632

[27] Bindea G, Mlecnik B, Tosolini M, Kirilovsky A, Waldner M, Obenauf AC, Angell H, Fredriksen T, Lafontaine L, Berger A, Bruneval P, Fridman WH, Becker C, et al. Spatiotemporal dynamics of intratumoral immune cells reveal the immune landscape in human cancer. Immunity. 2013; 39:782–95. 10.1016/j.immuni.2013.10.003 24138885

[28] Cully M. Chemical inhibitors make their RNA epigenetic mark. Nat Rev Drug Discov. 2019; 18:892–4. 10.1038/d41573-019-00179-5 31780844

[29] Shi S, Ma T, Xi Y. Characterization of the immune cell infiltration landscape in bladder cancer to aid immunotherapy. Arch Biochem Biophys. 2021; 708:108950. 10.1016/j.abb.2021.108950 34118215

[30] Wang S, Sun C, Li J, Zhang E, Ma Z, Xu W, Li H, Qiu M, Xu Y, Xia W, Xu L, Yin R. Roles of RNA methylation by means of N^6^-methyladenosine (m^6^A) in human cancers. Cancer Lett. 2017; 408:112–20. 10.1016/j.canlet.2017.08.030 28867248

[31] Song W, Yang K, Luo J, Gao Z, Gao Y. Dysregulation of USP18/FTO/PYCR1 signaling network promotes bladder cancer development and progression. Aging (Albany NY). 2021; 13:3909–25. 10.18632/aging.202359 33461172 PMC7906198

[32] Huang H, Weng H, Chen J. m^6^A Modification in Coding and Non-coding RNAs: Roles and Therapeutic Implications in Cancer. Cancer Cell. 2020; 37:270–88. 10.1016/j.ccell.2020.02.004 32183948 PMC7141420

[33] Rhea LP, Mendez-Marti S, Kim D, Aragon-Ching JB. Role of immunotherapy in bladder cancer. Cancer Treat Res Commun. 2021; 26:100296. 10.1016/j.ctarc.2020.100296 33421822

[34] Arfè A, Fell G, Alexander B, Awad MM, Rodig SJ, Trippa L, Schoenfeld JD. Meta-Analysis of PD-L1 Expression As a Predictor of Survival After Checkpoint Blockade. JCO Precis Oncol. 2020; 4:1196–206. 10.1200/PO.20.00150 35050777

[35] Chikina MD, Sealfon SC. Increasing consistency of disease biomarker prediction across datasets. PLoS One. 2014; 9:e91272. 10.1371/journal.pone.0091272 24740471 PMC3989170

[36] Feng ZH, Liang YP, Cen JJ, Yao HH, Lin HS, Li JY, Liang H, Wang Z, Deng Q, Cao JZ, Huang Y, Wei JH, Luo JH, et al. m6A-immune-related lncRNA prognostic signature for predicting immune landscape and prognosis of bladder cancer. J Transl Med. 2022; 20:492. 10.1186/s12967-022-03711-1 36309694 PMC9617388

[37] Yang J, Wu Z, Wu X, Chen S, Xia X, Zeng J. Constructing and validating of m6a-related genes prognostic signature for stomach adenocarcinoma and immune infiltration: Potential biomarkers for predicting the overall survival. Front Oncol. 2022; 12:1050288. 10.3389/fonc.2022.1050288 36620557 PMC9814967

[38] Zheng B, Wang J, Zhao G, Chen X, Yao Z, Niu Z, He W. A new m6A methylation-related gene signature for prognostic value in patient with urothelial carcinoma of the bladder. Biosci Rep. 2021; 41:BSR20204456. 10.1042/BSR20204456 33779704 PMC8035626

[39] Shulman Z, Stern-Ginossar N. The RNA modification N^6^-methyladenosine as a novel regulator of the immune system. Nat Immunol. 2020; 21:501–12. 10.1038/s41590-020-0650-4 32284591

[40] Lan Q, Liu PY, Haase J, Bell JL, Hüttelmaier S, Liu T. The Critical Role of RNA m^6^A Methylation in Cancer. Cancer Res. 2019; 79:1285–92. 10.1158/0008-5472.CAN-18-2965 30894375

[41] St Paul M, Ohashi PS. The Roles of CD8^+^ T Cell Subsets in Antitumor Immunity. Trends Cell Biol. 2020; 30:695–704. 10.1016/j.tcb.2020.06.003 32624246

[42] Raskov H, Orhan A, Christensen JP, Gögenur I. Cytotoxic CD8^+^ T cells in cancer and cancer immunotherapy. Br J Cancer. 2021; 124:359–67. 10.1038/s41416-020-01048-4 32929195 PMC7853123

[43] Bubeník J. Tumour MHC class I downregulation and immunotherapy (Review). Oncol Rep. 2003; 10:2005–8. 14534734

[44] Garrido F, Aptsiauri N, Doorduijn EM, Garcia Lora AM, van Hall T. The urgent need to recover MHC class I in cancers for effective immunotherapy. Curr Opin Immunol. 2016; 39:44–51. 10.1016/j.coi.2015.12.007 26796069 PMC5138279

[45] Seliger B, Cabrera T, Garrido F, Ferrone S. HLA class I antigen abnormalities and immune escape by malignant cells. Semin Cancer Biol. 2002; 12:3–13. 10.1006/scbi.2001.0404 11926409

[46] Kruse B, Buzzai AC, Shridhar N, Braun AD, Gellert S, Knauth K, Pozniak J, Peters J, Dittmann P, Mengoni M, van der Sluis TC, Höhn S, Antoranz A, et al. CD4^+^ T cell-induced inflammatory cell death controls immune-evasive tumours. Nature. 2023; 618:1033–40. 10.1038/s41586-023-06199-x 37316667 PMC10307640

[47] Cui C, Wang J, Fagerberg E, Chen PM, Connolly KA, Damo M, Cheung JF, Mao T, Askari AS, Chen S, Fitzgerald B, Foster GG, Eisenbarth SC, et al. Neoantigen-driven B cell and CD4 T follicular helper cell collaboration promotes anti-tumor CD8 T cell responses. Cell. 2021; 184:6101–18.e13. 10.1016/j.cell.2021.11.007 34852236 PMC8671355

[48] Zhou J, Zhang X, Hu J, Qu R, Yu Z, Xu H, Chen H, Yan L, Ding C, Zou Q, Ye Y, Wang Z, Flavell RA, Li HB. m^6^A demethylase ALKBH5 controls CD4^+^ T cell pathogenicity and promotes autoimmunity. Sci Adv. 2021; 7:eabg0470. 10.1126/sciadv.abg0470 34134995 PMC8208713

[49] Zheng Z, Zhang L, Cui XL, Yu X, Hsu PJ, Lyu R, Tan H, Mandal M, Zhang M, Sun HL, Sanchez Castillo A, Peng J, Clark MR, et al. Control of Early B Cell Development by the RNA N^6^-Methyladenosine Methylation. Cell Rep. 2020; 31:107819. 10.1016/j.celrep.2020.107819 32610122 PMC7371152

