# Supplementary material for: The identification of a N6-methyladenosin-modifed immune pattern to predict immunotherapy response and survival in urothelial carcinoma
Source: Aging (Albany NY). 2024 May 1;16(9):7774–98. doi: 10.18632/aging.205782 (PMC11131986; doi:10.18632/aging.205782)
Supplement: Supplementary Figures [file aging-16-205782-s001.pdf]

SUPPLEMENTARY FIGURES

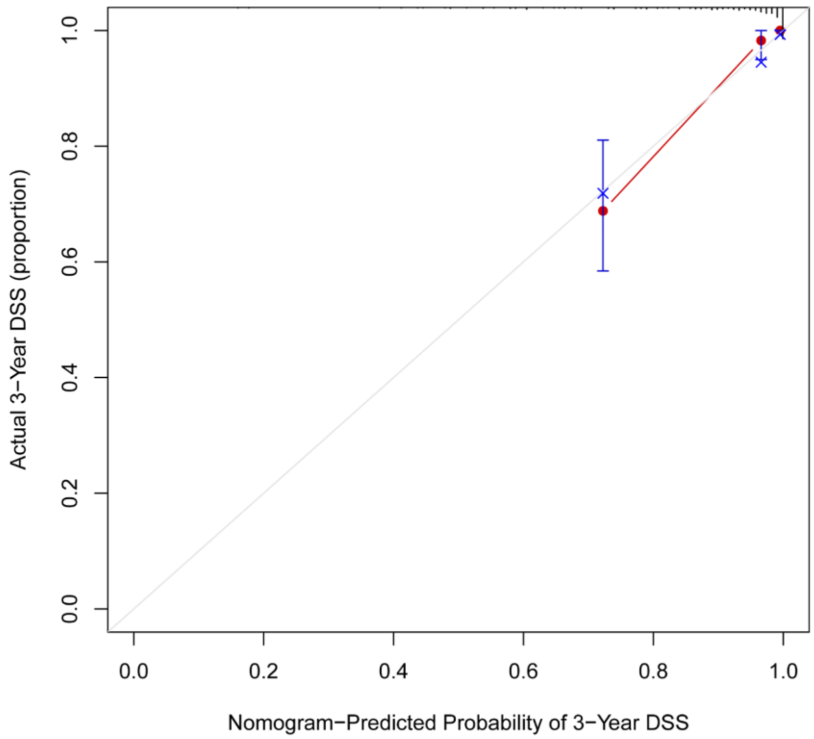

Supplementary Figure 1. Nomogram calibration curve used to predict the 3-year DSS period.

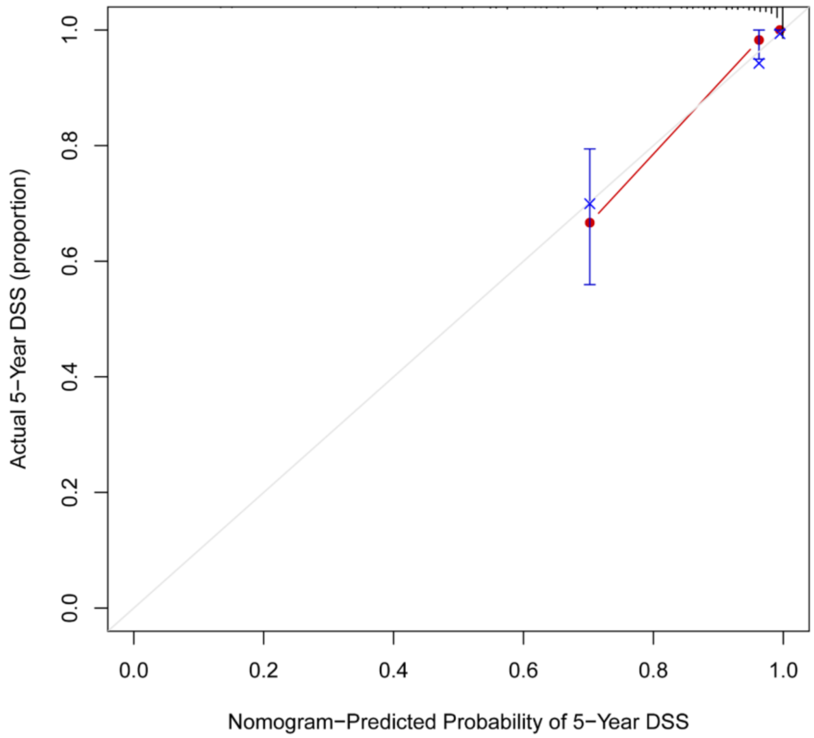

Supplementary Figure 2. Nomogram calibration curve used to predict the 5-year DSS period.

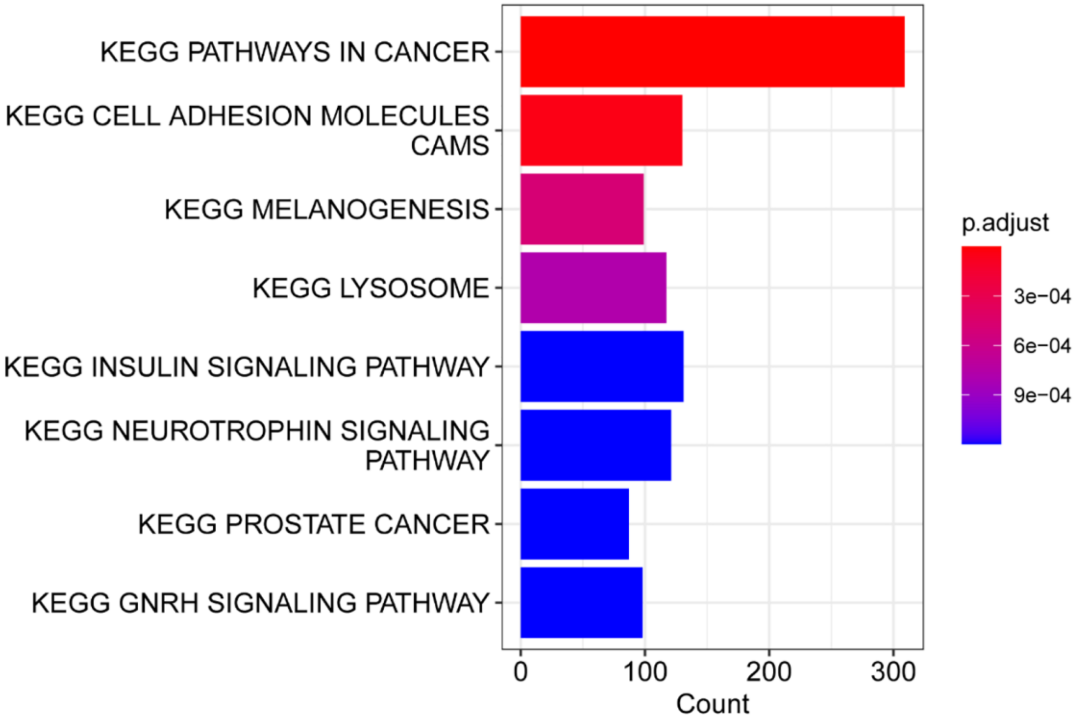

Supplementary Figure 3. Bar chart of significantly enriched KEGG pathways.
