# Supplementary material for: The identification of a N6-methyladenosin-modifed immune pattern to predict immunotherapy response and survival in urothelial carcinoma
Source: Aging (Albany NY). 2024 May 1;16(9):7774–98. doi: 10.18632/aging.205782 (PMC11131986; doi:10.18632/aging.205782)
Supplement: Supplementary Tables 3-6 and 8 [file aging-16-205782-s004.pdf]

SUPPLEMENTARY TABLES

Supplementary Table 3. The condition number of a matrix(K) of the PIGs in for data series.

Series	Condition number of a matrix(K)	If K < 100
**GSE32894**	14.27	Yes
**GSE32548**	13.59	Yes
**GSE13507**	7.09	Yes
**GSE48075**	9.74	Yes
**TCGA**	27.16	Yes

If K < 100, the degree of **collinearity** is very small; If 100 < = k < = 1000, there is a general degree of **collinearity**; If k > 1000, there is a serious **collinearity**.

Supplementary Table 4. Baseline information of 30 patients with UC.

Characteristic	Case number (percentage)
Gender	
Male	23 (76.7%)
Female	7 (23.3%)
Age	
<60	8 (26.7%)
<60	22 (73.3%)
Stage	
NMIBC (Ta/T1/Tis)	18 (60.0%)
MIBC (T2–T4)	12 (40.0%)
Disease Free Months	
<20	5 (16.7%)
≥20, <40	13 (43.3%)
≥40	12 (40%)

Supplementary Table 5. Primers used for quantitative real time-PCR.

**TNFRSF6B**	Forward primer	GTACGCGGAGTGGCAGAAA
	Reverse primer	CAGAGGACGTTGCAGTAGC
**CXCL2**	Forward primer	TCATAGCCACACTCAAGAAT
	Reverse primer	AGGAACAGCCACCAATAAG
**PSMD2**	Forward primer	TGCTCGTGGAACGACTAGG
	Reverse primer	CAGTTTGCCATAGTGTGGACG
**VEGFC**	Forward primer	GAGGAGCAGTTACGGTCTGTG
	Reverse primer	TCCTTTCCTTAGCTGACACTTGT
**GRB2**	Forward primer	CTGGGTGGTGAAGTTCAATTCT
	Reverse primer	GTTCTATGTCCCGCAGGAATATC
**CMTM1**	Forward primer	AACCTGAGTCATCCGAGGCA
	Reverse primer	CAGTCTCCGGTTGTTTCAAGT
**CBL**	Forward primer	TAGGCGAAACCTAACCAAACTG
	Reverse primer	AGAGTCCACTTGGAAAGATTCCT
**CYR61**	Forward primer	ACCGCTCTGAAGGGGATCT

	Reverse primer	ACTGATGTTTACAGTTGGGCTG
**SDC1**	Forward primer	ACGGCTATTCCCACGTCTC
	Reverse primer	TCTGGCAGGACTACAGCCTC
**CTSE**	Forward primer	CAGTCCAGCACATACAGCCA
	Reverse primer	GCTCCAATGATCCCGGACAA
**ACTIN**	Forward primer	CACCATTGGCAATGAGCGGTTC
	Reverse primer	AGGTCTTTGCGGATGTCCACGT

Supplementary Table 6. Information of 28 IRGs selected by RSH-VH.

Probe_ID	Symbol	ENSG_ID	Beta	HR (95% CI for HR)	Wald. test	*p*-value	Depth	rel. freq	Importance	EBTREZ_ ID
ILMN_1670918	INSR	ENSG00000171105	−1.6	0.21 (0.072–0.6)	8.3	0.0039	6.547	22	0.001	3643
ILMN_1814022	NR1H3	ENSG00000025434	−0.96	0.38 (0.23–0.64)	13	0.00028	6.003	23	0.00085	10062
ILMN_2387224	CTSE	ENSG00000196188	−0.93	0.4 (0.24–0.65)	13	0.00029	5.736	22	0.00289	1510
ILMN_1815308	SDC1	ENSG00000115884	−0.75	0.47 (0.31–0.71)	13	0.00026	6.395	22	0.00297	6382
ILMN_1799887	CTSE	ENSG00000196188	−0.63	0.53 (0.39–0.72)	17	3.4E-05	6.096	21	0.00625	1510
ILMN_2364384	PPARG	ENSG00000132170	−0.45	0.63 (0.5–0.8)	15	9.7E-05	6.941	22	0.00061	5468
ILMN_2355486	FAM3B	ENSG00000183844	−0.38	0.69 (0.59–0.81)	21	3.9E-06	6.875	26	0.00069	54097
ILMN_1787897	CXCL1	ENSG00000163739	0.34	1.4 (1.1–1.8)	7.9	0.005	7.136	22	−0.0001	2919
ILMN_2374449	SPP1	ENSG00000118785	0.35	1.4 (1.2–1.7)	12	0.00058	6.746	27	0.00046	6696
ILMN_1785699	PTHLH	ENSG00000087494	0.45	1.6 (1.2–2)	11	0.00073	6.673	21	0.0004	5744
ILMN_1774685	IL24	ENSG00000162892	0.45	1.6 (1.3–2)	15	8.8E-05	6.82	20	0.00083	11009
ILMN_1791726	TUBB3	ENSG00000258947	0.48	1.6 (1.3–2.1)	15	0.00013	6.618	21	0.00114	10381
ILMN_1682636	CXCL2	ENSG00000081041	0.49	1.6 (1.3–2.1)	17	4.7E-05	6.421	22	0.00135	2920
ILMN_2331231	TNFRSF6B	ENSG00000243509	0.56	1.8 (1.4–2.2)	24	1.2E-06	6.535	20	0.00125	8771
ILMN_2188264	CYR61/CNN1	ENSG00000142871	0.64	1.9 (1.4–2.5)	20	6.6E-06	6.104	24	0.00295	3491
ILMN_1696360	CTSB	ENSG00000164733	0.65	1.9 (1.2–3)	8	0.0046	6.873	21	0.00037	1508
ILMN_1691884	STC2	ENSG00000113739	0.66	1.9 (1.5–2.6)	21	4.6E-06	6.024	25	0.00066	8614
ILMN_1701204	VEGFC	ENSG00000150630	0.72	2 (1.3–3.1)	11	0.00077	5.99	21	0.00145	7424
ILMN_2382906	PLCG1	ENSG00000124181	0.85	2.3 (1.4–4.1)	9.2	0.0024	6.095	21	0.00099	5335
ILMN_1703178	SCG2	ENSG00000171951	0.91	2.5 (1.5–4)	14	0.00018	5.776	24	−0.0003	7857
ILMN_1758542	BMP1	ENSG00000168487	0.97	2.6 (1.7–4.1)	19	1.3E-05	6.722	22	0.0006	649
ILMN_2181968	CBL	ENSG00000110395	1	2.8 (1.3–5.9)	7.4	0.0063	6.309	24	0.00242	867
ILMN_1657968	MAP2K2	ENSG00000126934	1.1	2.9 (1.3–6.3)	6.8	0.0089	6.806	24	7.5E-05	5605
ILMN_1725814	IL24	ENSG00000162892	1.2	3.4 (1.7–7)	12	0.00065	6.851	21	2.4E-05	11009
ILMN_1748797	GRB2	ENSG00000177885	1.3	3.5 (1.6–7.6)	10	0.0016	6.539	22	0.00164	2885
ILMN_2328363	CMTM1	ENSG00000089505	1.3	3.8 (1.7–8.6)	11	0.0011	5.758	21	0.0022	113540
ILMN_2128128	SHFM1	ENSG00000127922	1.5	4.4 (1.4–14)	6.6	0.0099	6.167	21	0.00035	NA
ILMN_1712432	PSMD2	ENSG00000175166	1.9	6.6 (3.1–14)	23	1.5E-06	6.145	26	0.00135	5708
ILMN_1681515	CRLF1	ENSG00000006016	2	7.6 (2.1–28)	9.4	0.0022	6.328	22	0.00116	9244
ILMN_1707328	KLRC2	ENSG00000205809	5	150 (9.8–2300)	13	0.00032	6.556	21	3.3E-05	3822

Supplementary Table 8. Differential analysis of m6A modification peaks.

Symbol	logFC	*P*-value	Annotation	Gene ID	Transcript.ID	ENTREZ ID	GENENAME
INSR	1.439354567	0.000740458	3′UTR	ENSG00000171105.14	ENST00000601099.1	3643	insulin receptor
NR1H3	2.557239473	0.001460625	Promoter (≤1 kb)	ENSG00000025434.19	ENST00000495866.5	10062	nuclear receptor subfamily 1 group H

							member 3
CTSE	3.062398039	0.000505438	3′UTR	ENSG00000196188.12	ENST00000486757.1	1510	cathepsin E
SDC1	1.793082706	0.008954021	3′UTR	ENSG00000115884.11	ENST00000482879.5	6382	syndecan 1
PPARG	1.559761642	0.030422269	Promoter (2–3 kb)	ENSG00000132170.24	ENST00000682446.1	5468	peroxisome proliferator activated receptor gamma
FAM3B	1.978350189	0.035157454	Promoter (2–3 kb)	ENSG00000183844.17	ENST00000398646.3	54097	FAM3 metabolism regulating signaling molecule B
SPP1	2.465630299	0.00949005	Promoter (≤1 kb)	ENSG00000118785.15	ENST00000505146.1	6696	secreted phosphoprotein 1
CXCL2	2.773177463	0.003233683	Promoter (≤1 kb)	ENSG00000081041.9	ENST00000508487.3	2920	C-X-C motif chemokine ligand 2
CTSB	1.459450688	0.002211906	3′UTR	ENSG00000164733.23	ENST00000530290.5	1508	cathepsin B
PLCG1	2.46654589	4.95E-05	3′UTR	ENSG00000124181.15	ENST00000244007.7	5335	phospholipase C gamma 1
BMP1	1.781179922	0.016151302	3′UTR	ENSG00000168487.20	ENST00000522332.1	649	bone morphogenetic protein 1
CBL	1.788575387	0.029705874	Promoter (≤1 kb)	ENSG00000110395.8	ENST00000637974.1	867	Cbl proto-oncogene
MAP2K2	1.999922514	0.02765859	Exon (ENST00000262948.10/ ENSG00000126934.15, exon 3 of 11)	ENSG00000126934.15	ENST00000602167.5	5605	mitogen-activated protein kinase kinase 2
GRB2	2.137806528	0.009303183	Promoter (1–2 kb)	ENSG00000177885.15	ENST00000581959.1	2885	growth factor receptor bound protein 2
CMTM1	2.946544842	0.001246492	Promoter (≤1 kb)	ENSG00000089505.18	ENST00000332695.11	113540	CKLF like MARVEL transmembrane domain containing 1
PSMD2	2.368671003	0.004369637	Promoter (≤1 kb)	ENSG00000175166.17	ENST00000445558.6	5708	proteasome 26S subunit ubiquitin receptor, non-ATPase 2
CRLF1	1.799564651	0.035473845	Promoter (≤1 kb)	ENSG00000006016.12	ENST00000594325.1	9244	cytokine receptor like factor 1
